# Mitochondria and lipid raft-located F_O_F_1_-ATP synthase as major therapeutic targets in the antileishmanial and anticancer activities of ether lipid edelfosine

**DOI:** 10.1371/journal.pntd.0005805

**Published:** 2017-08-22

**Authors:** Janny A. Villa-Pulgarín, Consuelo Gajate, Javier Botet, Alberto Jimenez, Nicole Justies, Rubén E. Varela-M, Álvaro Cuesta-Marbán, Ingrid Müller, Manuel Modolell, José L. Revuelta, Faustino Mollinedo

**Affiliations:** 1 Instituto de Biología Molecular y Celular del Cáncer, Centro de Investigación del Cáncer, Consejo Superior de Investigaciones Científicas (CSIC)-Universidad de Salamanca, Campus Miguel de Unamuno, Salamanca, Spain; 2 Laboratory of Cell Death and Cancer Therapy, Department of Cellular and Molecular Medicine, Centro de Investigaciones Biológicas, Consejo Superior de Investigaciones Científicas (CSIC), Madrid, Spain; 3 Metabolic Engineering Group, Departamento de Microbiología y Genética, Universidad de Salamanca, Edificio Departamental, Campus Miguel de Unamuno, Salamanca, Spain; 4 Department of Cellular Immunology, Max-Planck-Institut für Immunbiologie und Epigenetik, Freiburg, Germany; 5 Department of Medicine, Section of Immunology, St. Mary’s Campus, Imperial College London, London, United Kingdom; CSIR-Indian Institute of Chemical Biology, INDIA

## Abstract

**Background:**

Leishmaniasis is the world’s second deadliest parasitic disease after malaria, and current treatment of the different forms of this disease is far from satisfactory. Alkylphospholipid analogs (APLs) are a family of anticancer drugs that show antileishmanial activity, including the first oral drug (miltefosine) for leishmaniasis and drugs in preclinical/clinical oncology trials, but their precise mechanism of action remains to be elucidated.

**Methodology/Principal findings:**

Here we show that the tumor cell apoptosis-inducer edelfosine was the most effective APL, as compared to miltefosine, perifosine and erucylphosphocholine, in killing *Leishmania* spp. promastigotes and amastigotes as well as tumor cells, as assessed by DNA breakdown determined by flow cytometry. In studies using animal models, we found that orally-administered edelfosine showed a potent *in vivo* antileishmanial activity and diminished macrophage pro-inflammatory responses. Edelfosine was also able to kill *Leishmania* axenic amastigotes. Edelfosine was taken up by host macrophages and killed intracellular *Leishmania* amastigotes in infected macrophages. Edelfosine accumulated in tumor cell mitochondria and *Leishmania* kinetoplast-mitochondrion, and led to mitochondrial transmembrane potential disruption, and to the successive breakdown of parasite mitochondrial and nuclear DNA. Ectopic expression of Bcl-X_L_ inhibited edelfosine-induced cell death in both *Leishmania* parasites and tumor cells. We found that the cytotoxic activity of edelfosine against *Leishmania* parasites and tumor cells was associated with a dramatic recruitment of F_O_F_1_-ATP synthase into lipid rafts following edelfosine treatment in both parasites and cancer cells. Raft disruption and specific F_O_F_1_-ATP synthase inhibition hindered edelfosine-induced cell death in both *Leishmania* parasites and tumor cells. Genetic deletion of F_O_F_1_-ATP synthase led to edelfosine drug resistance in *Saccharomyces cerevisiae* yeast.

**Conclusions/Significance:**

The present study shows that the antileishmanial and anticancer actions of edelfosine share some common signaling processes, with mitochondria and raft-located F_O_F_1_-ATP synthase being critical in the killing process, thus identifying novel druggable targets for the treatment of leishmaniasis.

## Author summary

Leishmaniasis is a major health problem worldwide, and can result in loss of human life or a lifelong stigma because of bodily scars. According to World Health Organization, leishmaniasis is considered as an emerging and uncontrolled disease, and its current treatment is far from ideal, with only a few drugs available that could lead to drug resistance or cause serious side-effects. Here, we have found that mitochondria and raft-located F_O_F_1_-ATPase synthase are efficient druggable targets, through which an ether lipid named edelfosine exerts its antileishmanial action. Edelfosine effectively kills *Leishmania* spp. promastigotes and amastigotes. Our experimental animal models demonstrate that oral administration of edelfosine exerts a potent antileishmanial activity, while inhibits macrophage pro-inflammatory responses. Our results show that both *Leishmania* and tumor cells share mitochondria and raft-located F_O_F_1_-ATPase synthase as major druggable targets in leishmaniasis and cancer therapy. These data, showing a potent antileishmanial activity of edelfosine and unveiling its mechanism of action, together with the inhibition of the inflammatory responses elicited by macrophages, suggest that the ether lipid edelfosine is a promising oral drug for leishmaniasis, and highlight mitochondria and lipid raft-located F_O_F_1_-ATP synthase as major therapeutic targets for the treatment of this disease.

## Introduction

Leishmaniasis, caused by several species of the protozoan *Leishmania* parasite, is one of the world’s most neglected diseases in terms of drug research and development, and for which current therapy is not satisfactory [1]. At present, about 350 million people in 98 countries worldwide are at risk of contracting leishmaniasis, and some 0.9–1.6 million new cases occur yearly, with about 0.7–1.2 million cases of self-healing cutaneous leishmaniasis, which can result in disfiguring skin lesions, and 0.2–0.4 million cases per year of life-threatening visceral leishmaniasis, which is a fatal disease if left untreated [1–3]. Leishmaniasis is the world’s second-deadliest parasitic disease after malaria, with a tentative estimate of 20,000 to 40,000 leishmaniasis deaths occurring annually [3], and has been classed as a category 1 disease (“emerging and uncontrolled”) by the World Health Organization (WHO). At present there are very few available antileishmanial drugs, being in general toxic, and the first line drugs are becoming ineffective due to emerging drug resistance [1, 2]. Thus, the development of novel therapeutic drugs is urgently needed. Leishmaniasis is transmitted by the bite of a female sandfly vector (*Lutzomyia* in the Americas and *Phlebotomus* elsewhere) infected with *Leishmania* parasites. Infection of humans and other animal hosts is initiated by flagellated promastigotes that develop within the digestive tract of the sandfly vector and are injected during a sandfly blood meal. Promastigotes are internalized into a number of phagocytic host cells, including neutrophils, dendritic cells, and macrophages, but proliferate only within the macrophage as aflagellate amastigotes [4, 5].

**Fig 1:**
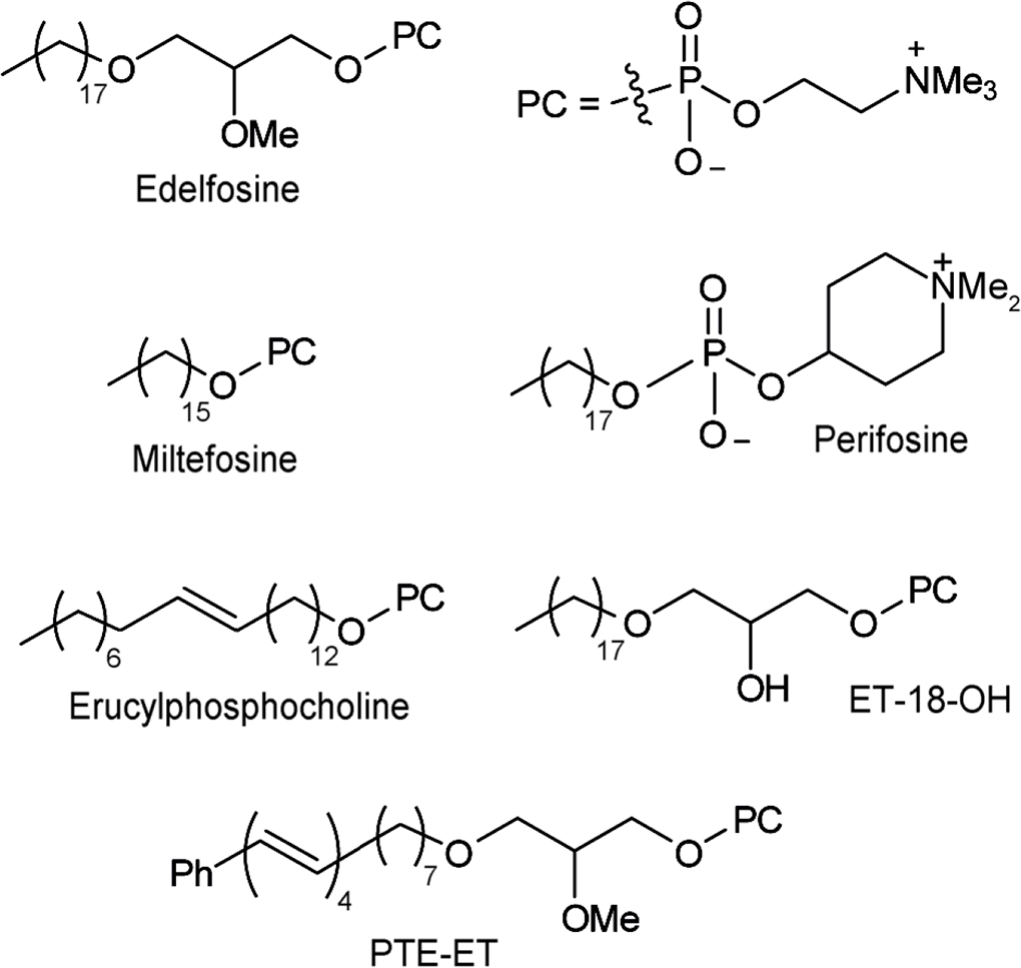
Chemical structures of APLs and edelfosine fluorescent analog PTE-ET used in this manuscript. The chemical structure of phosphatidylcholine (PC) is also shown. Me, methyl group.

The so-called alkylphospholipid analogs (APLs) constitute a class of structurally-related antitumor compounds with multiple therapeutic indications, and include a number of clinically relevant and/or promising drugs, such as miltefosine (hexadecylphosphocholine), edelfosine (1-*O*-octadecyl-2-*O*-methyl-*rac*-glycero-3-phosphocholine), perifosine (octadecyl (1,1-dimethyl-piperidinio-4-yl)-phosphate) and erucylphosphocholine ((13Z)-docos-13-en-1-yl 2-(trimethylammonio)ethyl phosphate) (ErPC) (Fig 1) [6–8]. So far, miltefosine is the only APL that has entered the clinic, registered as Impavido, the first orally-effective treatment for visceral leishmaniasis, and as Miltex, a topical chemotherapy and palliative treatment in cutaneous metastases from breast cancer [7, 9, 10]. APLs induce an apoptosis-like cell death in *Leishmania* parasites [11, 12], but their antiparasitic mechanism of action remains unknown, although lipid metabolism [13] and dramatic increases in membrane dynamics [14] have been suggested to play a role.

The ether lipid edelfosine induces apoptosis in tumor cells, involving cholesterol-rich lipid rafts and mitochondria [15–20]. Lipid rafts are membrane microdomains highly enriched in cholesterol and sphingolipids, and recent findings in mammalian cells suggest that lipid rafts act as death-promoting scaffolds, where Fas/CD95 and downstream signaling molecules are recruited to tigger apoptosis [21–23]. Raft domains have also been described in *Leishmania* spp., although their biochemical and functional characterization remains incomplete [24].

Here, we analyzed whether our knowledge on processes involved in the anticancer activity of APLs could provide some insight into their antileishmanial mechanism of action. In addition, we tested the effect of APLs on intact and *Leishmania*-infected macrophages as the main host cells for *Leishmania* parasites, as well as the *in vivo* antileishmanial activity of edelfosine in animal models. In this study, we report the existence of common mechanisms that underlie the antileishmanial and antitumor activities of the APL edelfosine, involving mitochondria, lipid rafts and F_O_F_1_-ATP synthase (also named as F_O_F_1_-ATPase), which might open up new avenues for the development of novel targeted therapies.

## Materials and methods

### Ethics statement

Animal procedures were approved by the institutional research commission of the University of Salamanca, and were approved by the Ethics Committee of the University of Salamanca (protocol approval number 48531). Animal procedures complied with the Spanish (Real Decreto RD1201/05) and the European Union (European Directive 2010/63/EU) guidelines on animal experimentation for the protection and humane use of laboratory animals, and were conducted at the accredited Animal Experimentation Facility (Servicio de Experimentación Animal) of the University of Salamanca (Register number: PAE/SA/001).

### Drugs and reagents

Edelfosine was from R. Berchtold (Biochemisches Labor, Bern, Switzerland). Miltefosine was from Calbiochem (Cambridge, MA). Perifosine and erucylphosphocholine were from Zentaris (Frankfurt, Germany). Rotenone, malonate, antimycin A, azide, oligomycin and CCCP were from Sigma (St. Louis, MO).

### Cells and culture conditions

The *Leishmania* strains used in this study were: *L*. *donovani* (MHOM/IN/80/DD8), *L*. *major* LV39 (MRHO/SU/59/P), *L*. *panamensis* (MHOM/CO/87/UA140), *L*. *infantum* (MCAN/ES/96/BCN/150, MON-1), kindly provided by Iván D. Vélez (Programa de Estudio y Control de Enfermedades Tropicales–PECET-, Medellin, Colombia), Ingrid Müller (Imperial College London, London, UK) and Antonio Jiménez-Ruiz (Universidad de Alcalá, Alcalá de Henares, Spain). To visualize *Leishmania* parasites inside macrophages we used GFP-*L*. *panamensis* promastigotes [25, 26]. *Leishmania* promastigotes were grown at 26°C in RPMI-1640 culture medium (Invitrogen, Carlsbad, CA), supplemented with 10% fetal bovine serum (FBS), 2 mM L-glutamine, 100 U/ml penicillin, and 100 μg/ml streptomycin. Promastigotes were treated at 26°C with the indicated compounds during their logarithmic growth phase (1.5 x 10^6^ parasites/ml). Late stationary promastigotes were obtained after incubation of the parasites for more than 6 days with a starting inoculum of 1 x 10^6^ parasites/ml. *Leishmania* axenic amastigotes were obtained as previously described [27]. Human acute T-cell leukemia Jurkat (American Type Culture Collection, Manassas, VA), myeloid leukemia HL-60 (American Type Culture Collection), multiple myeloma MM144 (provided by A. Pandiella, CIC, IBMCC, Salamanca, Spain), and cervical carcinoma HeLa **(**American Type Culture Collection) cell lines, as well as the mouse macrophage cell line J774 (American Type Culture Collection) were grown in RPMI-1640 culture medium supplemented with 10% FBS, 2 mM L-glutamine, 100 U/ml penicillin, and 100 μg/ml streptomycin at 37°C in humidified 95% air and 5% CO_2_.

### Yeast experiments and growth conditions

*Saccharomyces cerevisiae* yeast (BY4741 strain: MAT**a** *his3*Δ*1 leu2*Δ*0 met15*Δ*0 ura3*Δ*0*) was grown on standard synthetic complete medium (SDC), which consisted of synthetic minimal medium (SD; 0.17% yeast nitrogen base without amino acids, 2% glucose and supplements according to the requirements of the strains) with 0.079% complete supplement mixture (ForMedium, Norwich, UK). Yeast cultures were incubated at 30°C, and growth of cells untreated or treated with edelfosine was monitored by optical density at a wavelength of 595 nm (OD_595_). Cells were incubated for the indicated times and sample aliquots were taken to measure absorbance at 595 nm. Edelfosine was used at the concentrations indicated in the corresponding figure in liquid medium. The *atp7*Δ mutant was obtained from the EUROSCARF haploid deletion library in the BY4741 background [28]. This *atp7*Δ mutant was complemented with the corresponding wild-type gene expressed from a centromeric plasmid (pRS416), and yeast growth was determined as above.

### Bcl-X_L_ transfection

*L*. *infantum* transfected with pX63-Neo or pX63-*bcl-x*_*L*_ were kindly provided by Antonio Jiménez-Ruiz (Universidad de Alcalá, Alcalá de Henares, Spain) and grown in medium containing 100 μg/ml G418 (Sigma) [29]. HeLa cells (1–2 x 10^5^) were transfected with 2 μg of pSFFV-*bcl-x*_*L*_ or pSFFV-Neo expression vector [15], using Lipofectin reagent (Life Technologies, Carlsbad, CA). Transfected clones were selected by growth in the presence of 500 μg/ml G418, and monitored by Western blotting using the 2H12 anti-29 kDa Bcl-X_L_ monoclonal antibody (BD Biosciences PharMingen, San Diego, CA).

### Generation of mouse bone marrow-derived macrophages (BMM)

Murine bone marrow cells were obtained by flushing out the femurs of mice from (C57BL/6 x BALB/c)F1 (CBF1) mice and cultured as previously described [30] in hydrophobic Teflon bags (Biofolie 25, Heraeus, Hanau, Germany) with DMEM culture medium containing 10% FBS, 5% horse serum, 2 mM L-glutamine, 60 μM 2-mercaptoethanol, 1 mM sodium pyruvate, 1% non-essential amino acids, 100 U/ml penicillin, 100 μg/ml streptomycin, and the supernatant of L929 fibroblasts at a final concentration of 15% (v/v) as a source of colony-stimulating factors which drive cells towards a >95% pure BMM [31].

### BMM obtained from edelfosine-treated mice

CBF1 mice were treated orally with edelfosine (5 mg/kg body weight), daily for 13 days in 100 μl PBS, and then BMM were prepared as above. No weight loss or other visible side-effects were observed in mice treated with edelfosine.

### Macrophage cell viability assay

Cell viability at the indicated times was measured by the WST-1 reduction to formazan method (Roche Diagnostics, Basel, Switzerland). 10^5^ cells were incubated for 2 h at 37°C with 10 μl WST-1 solution in 0.2 ml DMEM culture medium supplemented with 10% FBS in a flat-bottom microtitre plate, and then absorbance was determined at 440 nm.

### Determination of superoxide anion

The production of superoxide anion (2 x 10^5^ cells in 0.2 ml Hepes-DMEM without pH indicator and containing 125 μM lucigenin, 37°C) was initiated by addition of 50 μg zymosan, and measured as lucigenin-dependent chemiluminescence using a Microlumat LB96P (Berthold, Wildbad, Germany) [32].

### Nitric oxide (NO) determination

NO end product nitrite was measured using the Griess reagent as previously described [32]. Culture supernatant was mixed with 100 μl of 1% sulphanilamide, 0.1% N-(1-naphthyl)ethylenediamine dihydrochloride and 2.5% H_3_PO_4_. Absorbance was measured at 540 nm in a microplate reader (Molecular Devices, Ismaning, Germany). LPS from *S*. *abortus equi* was kindly provided by Chris Galanos (Max-Planck-Institut, Freiburg, Germany).

### IFN-γ assay

IFN-**γ** was determined by a commercially available (Pharmingen) sandwich ELISA test according to the manufacturer’s protocol.

### Determination of intracellular ATP level

The ATP content was determined by the luciferin–luciferase method [33]. The assay is based on the requirement of luciferase for ATP in producing light (emission maximum 560 nm at pH 7.8). Briefly, cells (2 x 10^6^) were harvested after treatment, resuspended in 1X PBS, and assayed for ATP using the Molecular Probes ATP determination kit (Thermo Fisher Scientific, Waltham, MA). The amount of ATP in each experimental sample was calculated from a standard curve prepared with ATP and expressed as percentage of the amount of ATP found in untreated control cells.

### Analysis of apoptosis-like cell death by flow cytometry

1.5 x 10^6^ *Leishmania* spp. promastigotes or axenic amastigotes, and 10^6^ Jurkat cells or other human cells were incubated in the absence or presence of the indicated concentrations of APLs for different incubation times, and then analyzed for DNA breakdown by flow cytometry, using a fluorescence-activated cell sorting (FACS)Calibur flow cytometer (Becton Dickinson, San Jose, CA), as previously described [16]. Quantitation of apoptotic-like cells was monitored following cell cycle analysis as the percentage of cells in the sub-G_0_/G_1_ region, representing hypodiploids or apoptotic-like cells [16].

### Cytofluorimetric analysis of mitochondrial transmembrane potential (ΔΨ_m_) and generation of reactive of oxygen species (ROS)

2 x 10^6^ *Leishmania* parasites and 10^6^ Jurkat cells were pelleted by centrifugation, washed with PBS, incubated in 1 ml PBS containing 20 nM 3,3′-dihexyloxacarbocyanine-iodide (DiOC_6_(3), green fluorescence; Molecular Probes, Leiden, The Netherlands) and 2 μM dihydroethidine (HE, red fluorescence after oxidation; Sigma) at room temperature and darkness for 20 min, and then analyzed on a Becton Dickinson FACSCalibur flow cytometer as previously described [16].

### Macrophage infection

Macrophages, cultured in RPMI 1640 culture medium containing 10% FBS, were incubated for 4 h with stationary-phase *L*. *panamensis* promastigotes at a 10:1 parasite-to-macrophage ratio. Then, cell monolayers were extensively washed and incubated in complete culture medium with or without edelfosine for 24 h. The intracellular parasite load was calculated by limiting dilution assay as previously reported [34]. Alternatively, macrophage monolayers infected with green fluorescent protein (GFP)-expressing p6.5-*egfp*-*L*. *panamensis* parasites were cultured in glass coverslips placed into culture vessels (Corning, Lowell, MA). After 24 h, coverslips were washed, and the rate of intracellular amastigotes and infected macrophages was visualized using a fluorescence microscope. Results are shown as the percentage of infected macrophages and as the parasite/macrophage ratio after counting 100 macrophages.

### *In vivo* antileishmanial activity

Four-week-old male Syrian golden hamsters (*Mesocricetus auratus*) (about 120 g) were obtained from Charles River Laboratories (Lyon, France) and maintained in a pathogen-free facility. Animals were handled according to institutional guidelines, complying with the Spanish legislation, in an animal room with 12-h light/dark cycle at a temperature of 22°C, and received a standard diet and water *ad libitum*. Hamsters were inoculated intradermally in the nose with 1 x 10^6^ stationary-phase promastigotes in a volume of 50 μl PBS and treated with a daily oral administration of edelfosine (20 mg/kg in water), or an equal volume of vehicle (water) as previously described [26]. Nose swelling was evaluated through weekly caliper measurements, and compared with the nose size before inoculation and treatment. Evolution index of the lesion was calculated as the size (mm) of the lesion during treatment/size of the lesion before treatment. No loss in animal body weight and no sign of morbidity were detected during the 28-day drug treatment, and animals were killed, following institutional guidelines, 24 h after the last drug administration. Parasite burden in the infected tissues was calculated by limiting dilution assay as previously described [26].

### TUNEL assay

Apoptosis-like cell death was also analyzed *in situ* by the TUNEL technique using the Fluorescein Apoptosis Detection System (Promega, Madison, WI), according to the manufacturer’s instructions. Parasites were fixed with 4% formaldehyde for 20 min on microscope slides, permeabilized with 0.2% Triton X-100, stained for fragmented DNA using the above kit, and then propidium iodide was added for 15 min to stain both apoptotic-like and intact cells as previously described [17, 35, 36]. Propidium iodide stained all cells in red, whereas fluoresecin-12-dUTP was incorporated at the 3’-OH ends of fragmented DNA, resulting in localized green fluorescence within the nucleus of apoptotic-like cells. Samples were analyzed with a Zeiss LSM 510 laser scan confocal microscope (Carl Zeiss AG, Jena, Germany).

### Edelfosine localization by fluorescence microscopy

*L*. *panamensis* and HeLa cells were treated for 1 h with 10 μM fluorescent edelfosine analog *all*-(*E*)-1-*O*-(15’-phenylpentadeca-8’,10’,12’,14’-tetraenyl)-2-*O*-methyl-*rac*-glycero-3-phosphocholine (PTE-ET) (Fig 1), kindly provided by F. Amat-Guerri and A.U. Acuña (Consejo Superior de Investigaciones Cientificas, Madrid, Spain) as described [17, 36, 37], and then incubated with 100 nM cell-permeant MitoTracker probe (Molecular Probes) for 20 min to label mitochondria. Colocalization was analyzed by excitation of the corresponding fluorochromes in the same section of samples, using a fluorescence microscope (Axioplan 2; Carl Zeiss MicroImaging, Inc., Oberkochen, Germany) and a digital camera (ORCA-ER-1394; Hamamatsu, Hamamatsu City, Japan).

### Disruption of lipid microdomains in *Leishmania* promastigotes and Jurkat cells

Parasites (2 x 10^6^/ml) were incubated in serum-free medium with 2.5 mg/ml methyl-β-cyclodextrin (MCD) for 40 min at 26°C, and then washed 3 times with PBS, and resuspended in complete culture before edelfosine addition. For cholesterol depletion in Jurkat cells, 2.5 x 10^5^ cells/ml were incubated with 2.5 mg/ml MCD for 30 min at 37°C in serum-free medium, and then washed 3 times with PBS, and resuspended in complete culture before edelfosine addition.

### Edelfosine uptake

Drug uptake was measured as previously described [15, 36], after incubating 2 x 10^6^ parasites or 10^6^ Jurkat cells with 10 nmol [^3^H]edelfosine (10 μM) (Amersham Buchler, Braunschweig, Germany) for 1 h in RPMI-1640, 10% FBS, and subsequent washing (six times) with PBS + 2% BSA. [^3^H]edelfosine (specific activity, 42 Ci/mmol) was synthesized by tritiation of the 9-octadecenyl derivative (Amersham Buchler, Braunschweig, Germany).

### Lipid raft isolation

Lipid rafts were isolated from 1 x 10^8^ *Leishmania* promastigotes or 8×10^7^ Jurkat cells by using nonionic detergent lysis conditions and centrifugation on discontinuous sucrose gradients as previously reported [38, 39]. Twelve 1-ml fractions were collected from the top of the gradient, and 25 μl of each fraction were subjected to sodium dodecylsulfate (SDS)-polyacrylamide gel electrophoresis (PAGE) and assayed for the location of GM1-containing lipid rafts using the GM1-specific ligand cholera toxin (CTx) B subunit conjugated to horseradish peroxidase (Sigma, St. Louis, MO).

### Two-dimensional gel electrophoresis

The proteomic analysis was performed in the proteomics facility of Centro de Investigación del Cáncer (CIC), Salamanca, Spain, which belongs to ProteoRed, PRB2-ISCIII. Samples (100 μg protein) from pooled fractions enriched in lipid rafts (fractions 3–6 from the sucrose gradient) were precipitated with methanol/chloroform, and then the pellets were resuspended in rehydration buffer (7 M urea, 2M thiourea, 4% CHAPS, 50 mM DTT, 5 mM TCEP, 15 mg DeStreak, 0.5% IPG buffer). Samples were applied to 13 cm IPG strips with a nonlinear pH gradient of 3 to 10 (Amersham Biosciences). Isoelectric focusing was performed at 50 V for 12 hours, 500 V for 1 h, 1000 V for 1 h, a voltage gradient ranging from 1000 to 8000 V for 30 min, and finally 5 h until the voltage reached 35000 V. Strips were treated with SDS equilibration buffer (375 mM Tris-HCl pH 8.8, 6 M urea, 20% glycerol, 2% SDS) plus 2% DTT for 15 min for protein denaturation, and then with equilibration buffer plus 2.5% iodoacetamide for protein alkylation. The second dimension electrophoresis was performed on 10% SDS-polyacrylamide gels. The protein spots were visualized with Sypro Ruby Protein Gel Staining (Invitrogen, Carlsbad, CA).

### Spot excision, tryptic digestion of proteins, mass determination and protein identification

Spots of interest were automatically excised with Proteineer Spot Picker robotics workstation (Bruker Daltonics, Billerica, MA). The digestion was performed as previously described [40]. For MALDI-TOF peptide mass fingerprinting, a 0.5 μl aliquot of matrix solution (5 g/l 2,5-dihydroxybenzoic acid in 33% aqueous acetonitrile plus 0.1% trifluoroacetic acid) was manually loaded onto a 400 μm diameter AnchorChip Target plate (Bruker Analytic GmbH, Bremen, Germany) probe, and 1 μl of the above peptide extraction solution was added and allowed to dry at room temperature. Samples were analyzed on a Bruker Ultraflex MALDI-TOF mass spectrometer (Bruker-Franzen Analytic GmbH, Bremen, Germany). Each raw spectrum was opened in FlexAnalysis 3.0 (Bruker Daltonics) software and processed and analyzed using the following parameters: signal-to-noise threshold of 1, Savitzky-Golay algorithm for smoothing, tangential algorithm for baseline substraction, and centroid algorithm for monoisotopic peak assignment. In all cases, resolution was higher than 9000. The generated peaks were submitted to Mascot Server (version 2.2, February 2007) [41] using Bio Tools 3.1 (Bruker Daltonics) software, and searched against Uniprot database for human sequences and NCBI database for *Leishmania* sequences. Search parameters were set as follow: searches were restricted to all sequences for human searches and Other Eukaryota (69482 sequences) for *Leishmania* searches, up to one missed tryptic cleavage, mass accuracy of 100 ppm, MH+ monoisotopic masses, carbamidomethyl cysteine as fixed modification, and methionine oxidation as variable modification. Mowse scores with a value greater than 65 for human searches and 61 for *Leishmania* searches were considered as significant (p<0.05).

### Statistical analysis

Data are shown as mean ± SD. Between-group statistical differences were assessed using the Student’s *t* test. A *P*-value of <0.05 was considered statistically significant.

## Results

### Differential capacity of APLs in promoting cell death in *Leishmania* spp. and human cancer cells with apoptosis-like characteristics

**Fig 2:**
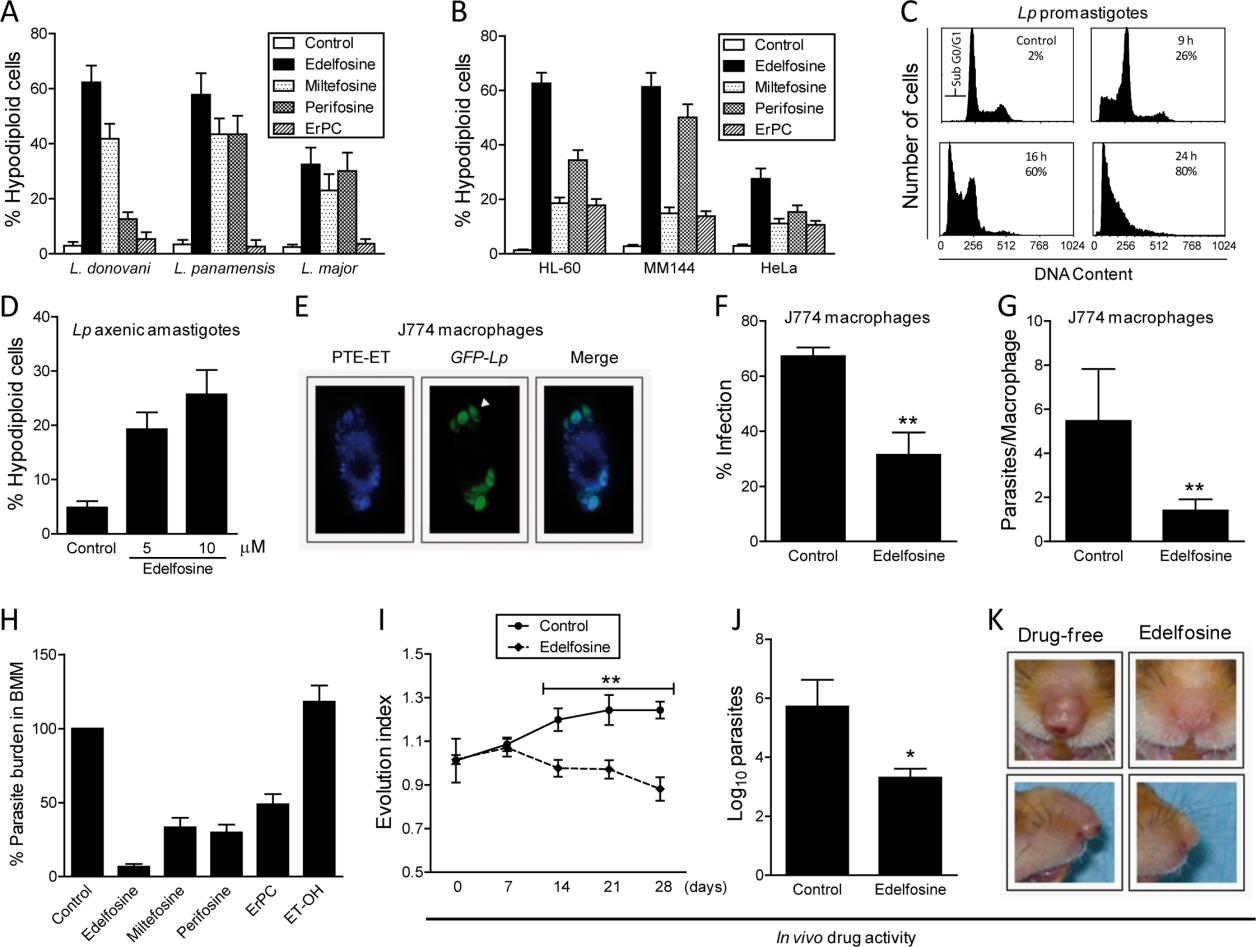
*In vitro* and *in vivo* antileishmanial activity of the antitumor ether phospholipid edelfosine. Apoptosis-like cell death was quantitated by flow cytometry as percentage of hypodiploid cells (sub-G_0_/G_1_) in different *Leishmania* spp. promastigotes (**A**) and human cancer cell lines (myeloid leukemia HL-60, multiple myeloma MM144, and cervical carcinoma HeLa) (**B**), following 24-h incubation with distinct APLs (10 μM). Untreated control cells were run in parallel. ErPC, erucylphosphocholine. (**C**) Time-course of 10 μM edelfosine-induced apoptosis-like cell death (% hypodiploid cells) in *L*. *panamensis* promastigotes. Untreated control parasites were run in parallel. (**D**) Induction of apoptosis-like cell death in *L*. *panamensis* axenic amastigotes treated with the indicated concentrations of edelfosine for 16 h. (**E**) GFP-*L*. *panamensis* (*GFP-Lp*)-infected J774 macrophages were incubated for 1 h with 10 μM PTE-ET and analyzed by fluorescence microscopy. Incubation of GFP-*Lp*-infected J774 macrophages with 10 μM edelfosine for 24 h decreased parasite infection (**F**) and the number of parasites per macrophage (**G**), as compared to untreated infected cells (Control). (**H**) Murine BMM were infected with *L*. *panamensis* and subsequently treated with distinct APLs (10 μM) or vehicle (Control). Parasite load was measured after 3-day incubation. (**I-K**) Hamsters were inoculated in the nose with *L*. *panamensis* promastigotes, and then treated orally with edelfosine (20 mg/kg, *n* = 8) or with water vehicle (Control) for 28 days. Evolution index during treatment (**I**) and parasite load in the nose at the end of the 4-week treatment (**J**) were determined. Edelfosine treatment dramatically reduced nose inflammation and damage at the end of treatment (**K**). Data shown are means ± SD or representative of three independent experiments performed. Asterisks indicate that the differences between control and edelfosine-treated groups are statistically significant. (*) *P*<0.05. (**) *P*<0.01.

First we analyzed the ability of different APLs (Fig 2A and 2B) in promoting apoptosis-like cell death in different *Leishmania* spp. promastigotes and human cancer cell lines, as assessed by DNA breakdown determined by flow cytometry. Our results showed that APLs ranked edelfosine > miltefosine ≥ perifosine > erucylphosphocholine (ErPC) for their leishmanicidal activity (Fig 2A), and edelfosine > perifosine > miltefosine ≅ erucylphosphocholine (ErPC) for their antitumor activity (Fig 2B), when incubated for 24 h at 10 μM with several *Leishmania* spp. promastigotes, including *L*. *donovani* (visceral leishmaniasis), *L*. *panamensis* (cutaneous and mucocutaneous leishmaniasis), and *L*. *major* (cutaneous leishmaniasis), or with human cancer cell lines, including myeloid leukemia HL-60 cells, multiple myeloma MM144 cells, and cervical cancer HeLa cells. This drug concentration (10 μM) corresponded to the pharmacologically relevant concentration range of edelfosine in plasma (10–20 μM), previously determined in a number of *in vivo* and pharmacokinetic studies [19, 42, 43]. We also found that edelfosine was very efficient in promoting cell death in additional human leukemic cell lines, including human T-cell acute lymphoblastic leukemia (T-ALL) cell lines Jurkat (53.4 ± 6.2% apoptosis) and CEM-C7H2 (58.2 ± 5.9% apoptosis). Edelfosine was equally effective against different *Leishmania* subgenera, including *Leishmania Leishmania* (*L*. *donovani*, *L*. *major*) and *Leishmania Viannia* (*L*. *panamensis*) (Fig 2A). The relative difference between the abilities to promote cell death of edelfosine *vs*. miltefosine was more evident using tumor cells than *Leishmania* spp. promastigotes, suggesting that processes involved in the mechanisms of action of both drugs are partially conserved, but not identical. For the ensuing studies, we focused our attention on the most effective compound, namely edelfosine, which has been considered as the APL prototype. Edelfosine induced DNA breakdown after 9-h incubation with *L*. *panamensis* promastigotes, and the percentage of parasites with a hypodiploid DNA content (sub-G_0_/G_1_ cell population) increased with the incubation time (Fig 2C), suggesting an apoptosis-like cell death in *Leishmania* parasites, similar to the apoptotic response triggered in cancer cells [15, 17, 35, 44]. Edelfosine (5 or 10 μM) also induced apoptosis-like cell death, as assessed by an increase in the sub-G_0_/G_1_ population, in *L*. *panamensis* axenic amastigotes (Fig 2D).

### Edelfosine accumulates into and kills *Leishmania* amastigotes in infected macrophages

Because *Leishmania* are obligate intracellular parasites that infect macrophages within the mammalian host, we examined the location of edelfosine in *L*. *panamensis*-infected J774 macrophage-like cells. We have previously found that mouse J774 macrophages were rather resistant to edelfosine [26], and 10 μM edelfosine was unable to mount an apoptotic response after 24-h incubation (<2.5% apoptosis). Using the blue-emitting fluorescent edelfosine analog PTE-ET (Fig 1), a *bona fide* compound to explore the subcellular localization of edelfosine [17, 19, 36, 37, 45], we found that it was mainly located into the parasites inside the macrophage (Fig 2E), which were visualized by using infective GFP-*L*. *panamensis* parasites [25]. Edelfosine treatment highly diminished the amount of infected J774 macrophages and the number of parasites per macrophage (Fig 2F and 2G). Following limiting dilution experiments, we found that edelfosine was the most effective APL, when compared to miltefosine, perifosine and erucylphosphocholine (ErPC), in killing *L*. *major* amastigotes in infected mouse BMM (Fig 2H). Edelfosine was highly dependent on its molecular structure for its antileishmanial activity, since a structurally related compound, ET-18-OH (1-*O*-octadecyl-*rac*-glycero-3-phosphocholine) (Fig 1), containing a hydroxyl group instead of the methoxy group at the C2 position, was unable to kill *Leishmania* protozoa (Fig 2H), similarly to what has been found in cancer cells [15, 44].

### *In vivo* antileishmanial activity of edelfosine

We have previously shown the potent antitumor activity of orally-administered edelfosine in different xenograft animal models [19, 43, 46]. Recently, we have also found that edelfosine was effective in the treatment of leishmaniasis in different animal models when used at 26 mg/kg body weight [26]. Here, we found that oral treatment of edelfosine at a lower dose (20 mg/kg body weight) exerted a potent *in vivo* antileishmanial activity in *L*. *panamensis*–infected golden hamsters (Fig 2I–2K), an appropriate animal model for reproducing the pathological features of human leishmaniasis [47]. *L*. *panamensis* promastigotes were inoculated into the nose of 16 golden hamsters, and then animals were randomly distributed into two cohorts of eight hamsters. Each cohort received a daily oral administration of edelfosine or water vehicle (control) for 28 days. Disease progression was monitored by nasal swelling, determined by serial caliper measurements, and ulceration. Oral treatment with edelfosine led to a dramatic decrease in nasal swelling and parasite load at the site of infection (Fig 2I–2K), and ameliorated the signs of leishmaniasis, leading to an almost normal morphologic appearance (Fig 2K).

### Edelfosine is taken up by macrophages and inhibits the generation of macrophage-derived pro-inflammatory mediators

**Fig 3:**
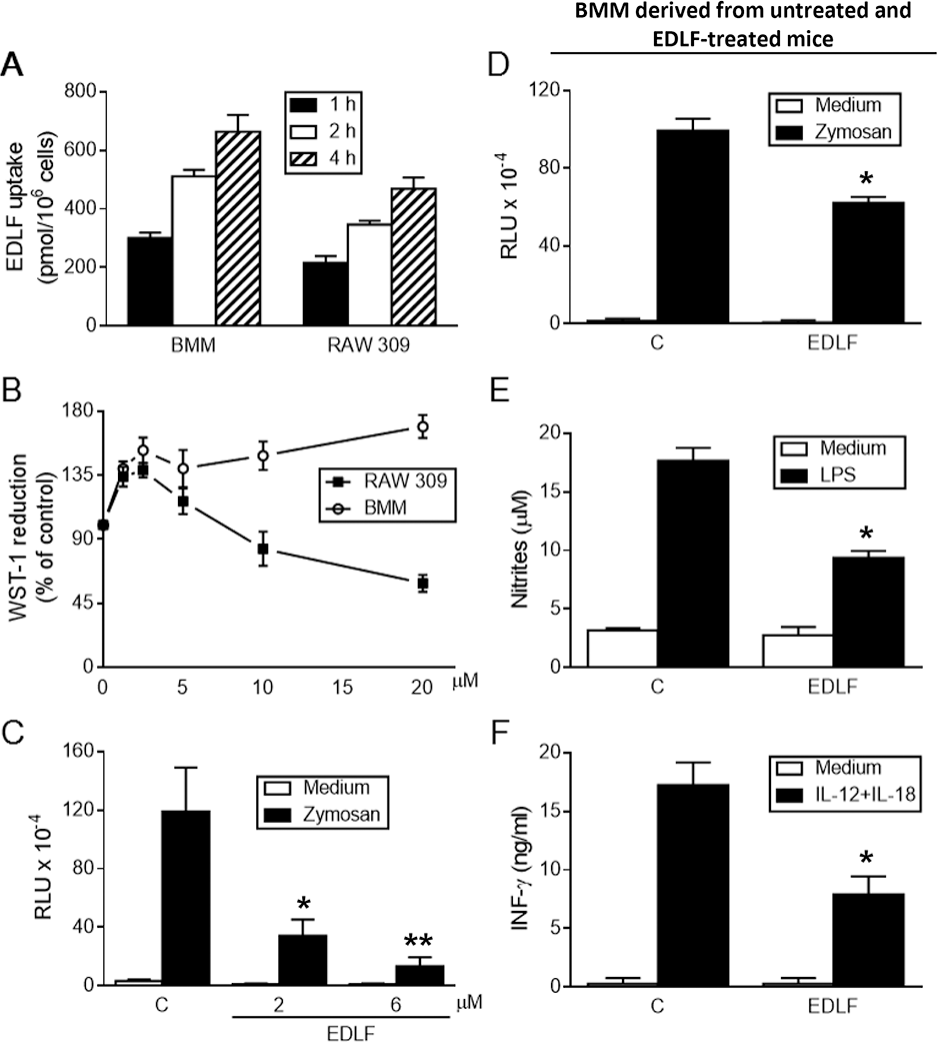
Edelfosine is taken up by macrophages and inhibits macrophage-derived inflammatory mediators. (**A**) Incorporation of edelfosine (EDLF) in mouse bone marrow-derived macrophages (BMM) and mouse RAW 309 Cr.1 tumor macrophage cell line. 10^6^ cells were incubated with 10 μM edelfosine (containing 0.05 μCi [^3^H]edelfosine) for the indicated times to measure drug uptake. (**B**) Edelfosine is cytotoxic for transformed macrophages but spares BMM. 2 x 10^6^ cells were incubated for 24 h in the absence or presence of the indicated concentrations of edelfosine (EDLF), and cytotoxicity was determined by the WST-1 reduction method. (**C**) Edelfosine inhibits superoxide anion generation in BMM. Superoxide anion was measured as lucigenin-dependent chemiluminescence (relative light units, RLU) in untreated control (C) or edelfosine (EDLF)-treated BMM that were incubated with medium alone or zymosan to induce the respiratory burst. (**D-F**) BMM from edelfosine-fed mice show a decreased generation of inflammatory mediators. BMM from untreated control mice (C) and from mice given orally edelfosine (EDLF) for two weeks were analyzed for their capacity to generate zymosan-induced superoxide anion (**D**), LPS-induced nitric oxide (**E**), and IL-12+IL-18-induced IFN-γ (**F**). Cells incubated with medium alone were run in parallel as a negative control of each assay. Data shown are means ± SD of five independent determinations. Asterisks indicate values that are significantly different from those of control mice (comparison between the black histograms of control and edelfosine-treated groups) at *P*<0.05 (*) and *P*<0.01 (**).

Edelfosine has been shown to be taken up preferentially by tumor cells, whereas normal non-malignant cells incorporated a relatively much lesser amount of the ether lipid [15, 17, 35]. Here, we found that normal mouse BMM took up large amounts of edelfosine, at even higher levels than the mouse RAW 309 Cr.1 tumor macrophage cell line (Fig 3A). However, edelfosine induced cell death in the transformed macrophage cell line, but spared BMM (Fig 3B). Interestingly, edelfosine blocked zymosan-induced respiratory burst in BMM (Fig 3C). Furthermore, BMM from mice that were orally treated with edelfosine (5 mg/kg body weight, daily) for two weeks showed a lower capacity to generate superoxide anion, NO and IL-12+IL-18-induced IFN-γ, when compared to BMM from mice treated with water vehicle (Fig 3D–3F). These results suggest that edelfosine treatment decreases macrophage pro-inflammatory responses. Our data, together with the above accumulation of edelfosine into the parasites in *Leishmania*-infected macrophages, suggest that edelfosine-induced killing of *Leishmania* is mediated by a direct action of the drug on the parasite, and not via generation of macrophage-derived antiparasitic molecules.

### Edelfosine induces kinetoplast DNA cleavage prior to nuclear DNA breakdown

**Fig 4:**
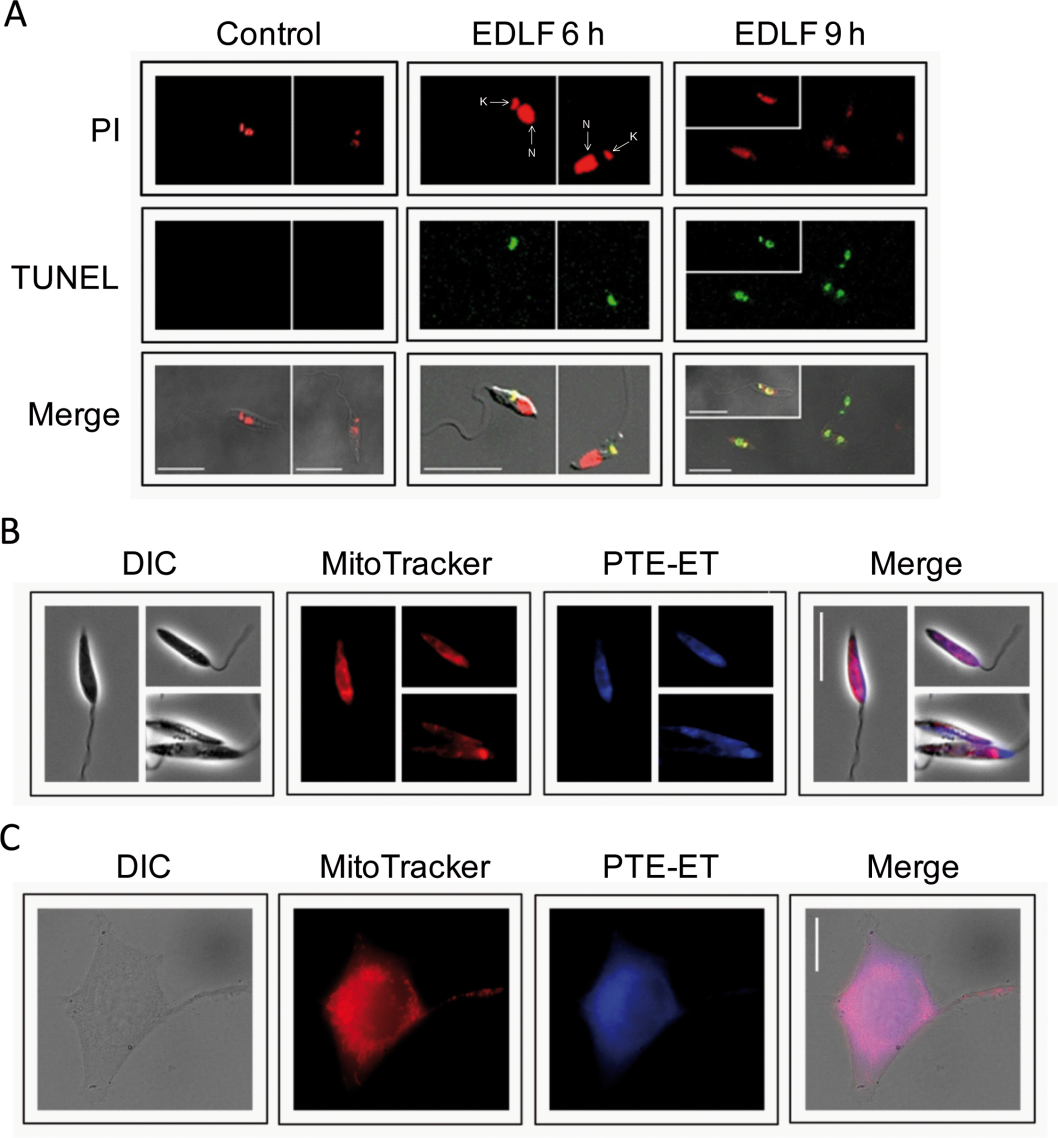
Edelfosine induces breakage of kinetoplast DNA prior to nuclear DNA breakdown, and accumulates in mitochondria in *Leishmania* parasites and cancer cells. (**A**) *L*. *panamensis* promastigotes were untreated (Control) or treated with 10 μM edelfosine (EDLF) for 6 and 9 h, and then analyzed by confocal microscopy for propidium iodide (PI) staining and TUNEL assay. The positions of the nucleus (N) and kinetoplast (K) are indicated by arrows. Merging of PI and TUNEL panels (Merge) shows the DNA-containing organelles with DNA disruption in yellow. The corresponding differential interference contrast (DIC) images were included in the Merge panels to highlight parasite morphology and facilitate kinetoplast identification. (**B**) *L*. *panamensis* promastigotes and (**C**) HeLa cancer cells were incubated with 10 μM PTE-ET (blue fluorescence) for 1 h, 100 nM MitoTracker (red fluorescence) for 20 min to localize mitochondria, and then analyzed by fluorescence microscopy. Areas of colocalization between mitochondria and PTE-ET in merge panels are purple. The corresponding differential interference contrast (DIC) images are also shown. Images are representative of three independent experiments. Bar, 20 μm.

DNA breakdown induced by edelfosine treatment in *Leishmania* was further assessed by the TUNEL assay, staining all cells in red through the binding of propidium iodide to DNA, and only those cells with fragmented DNA and free 3’-OH ends in green. Interestingly, we detected first kinetoplast-mitochondrial DNA degradation, followed by nuclear DNA fragmentation upon treatment of *L*. *panamensis* promastigotes with edelfosine (Fig 4A). These results suggest that the death process induced by edelfosine in *Leishmania* spp. parasites starts at the mitochondrial level.

### Edelfosine accumulates in the mitochondria of *Leishmania* and cancer cells

Next, we analyzed the subcellular localization of edelfosine in *Leishmania* promastigotes. The fluorescent edelfosine analog PTE-ET, which has been previously shown to fully mimic the antitumor [17, 19, 36, 37, 45, 48] and antileishmanial [49] actions of the parent drug edelfosine, accumulated mainly in the mitochondria of *L*. *panamensis* promastigotes, as indicated by colocalization with the specific mitochondrial marker MitoTracker (Fig 4B). PTE-ET also co-localized with MitoTracker-positive structures in human cervical carcinoma HeLa cells (Fig 4C).

### Mitochondrial involvement in edelfosine-induced *Leishmania* death

**Fig 5:**
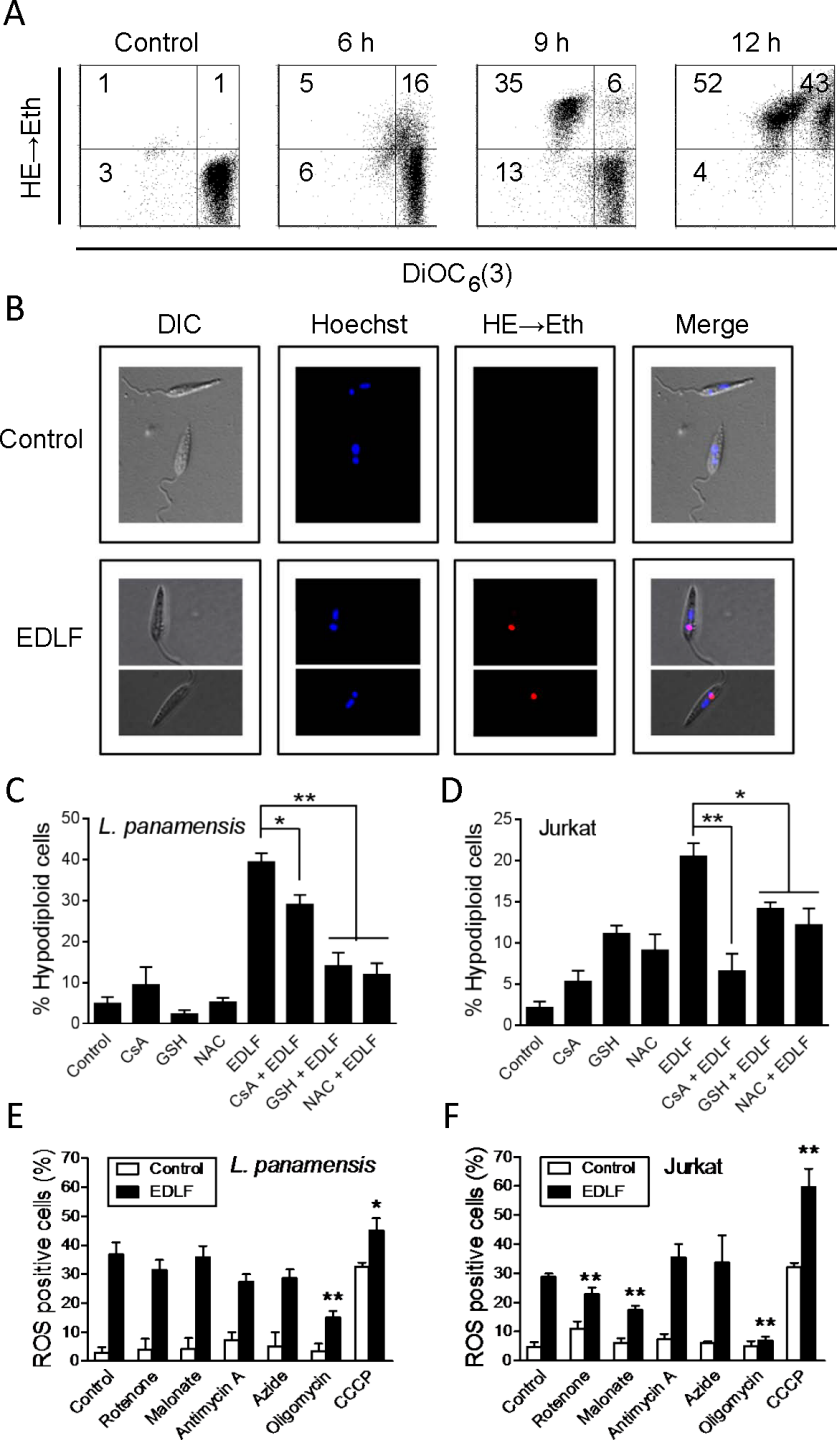
Involvement of mitochondria and ROS generation in edelfosine-induced cell death in *Leishmania* parasites and cancer cells. (**A**) *L*. *panamensis* promastigotes were untreated (Control) or treated with 10 μM edelfosine at the indicated times, and cells with disrupted ΔΨ_m_ (DiOC_6_(3)^low^) and ROS production (HE→Eth) were measured by flow cytometry. The numbers in each quadrant refer to the percentages of cells in each population. (**B**) *L*. *panamensis* promastigotes untreated (Control) and treated with edelfosine (EDLF) for 3 h were incubated with 2 μM HE and 10 μg/ml Hoechst 33342, and then analyzed by fluorescence microscopy. (**C**) *L*. *panamensis* promastigotes and (**D**) Jurkat cells were preincubated with 10 μg/ml CsA for 1 h, or with 10 mM NAC or 10 mM GSH for 2 h, and then incubated in the absence or presence of 10 μM edelfosine for 9 h. After treatment, the percentage of hypodiploid cells was analyzed by flow cytometry. Untreated control cells were run in parallel. (**E**) *L*. *panamensis* promastigotes and (**F**) Jurkat cells were preincubated with 10 μM rotenone, 5 mM malonate, 10 μM antimycin A, 1.5 mM azide, 50 μM CCCP or with 1 and 10 μM oligomycin (*L*. *panamensis* and Jurkat cells, respectively) for 1 h, and then incubated in the absence or presence of 10 μM edelfosine for 9 h. After treatment, cells producing ROS were quantified by flow cytometry. Untreated control cells were run in parallel. Data shown are means ± SD or representative of three independent experiments performed. Asterisks denote that the differences between the indicated groups (C and D) and with control cells (E and F) are statistically significant. (*) *P*<0.05. (**) *P*<0.01.

We next examined the time-course of the effect of edelfosine on the following mitochondrial-related processes in *L*. *panamensis* promastigotes: a) ROS generation, through the conversion of non-fluorescent dihydroethidine (HE) into red fluorescent ethidium (Eth) after its oxidation via ROS; and b) changes in ΔΨ_m_, through the accumulation of the fluorescent cationic probe DiOC6(3) (green fluorescence), which depends on the mitochondrial potential. As shown in Fig 5A, untreated parasites exhibited a high ΔΨ_m_ [(DiOC_6_(3))^high^], and the levels of intracellular ROS were undetectable [(HE → Eth)^low^]. Edelfosine induced first an increase in the percentage of cells with (HE → Eth)^high^, and then a loss in ΔΨ_m_ (Fig 5A). Changes in ROS generation and ΔΨ_m_ disruption apparently preceded DNA breakdown. Edelfosine induced Eth staining, i.e. ROS generation, in kinetoplasts, as assessed by using DNA staining to identify *L*. *panamensis* nuclei and kinetoplasts (mitochondrial DNA) (Fig 5B). The inhibitor of the mitochondrial permeability transition pore cyclosporin A [50], and the antioxidant agents glutathione (GSH) and N-acetylcysteine (NAC), inhibited edelfosine-induced cell death in *L*. *panamensis* promastigotes (Fig 5C). Likewise, cyclosporin A, GSH and NAC inhibited edelfosine-induced apoptosis in human T-cell leukemia Jurkat cells (Fig 5D). Taken together, our data suggest a critical role of mitochondria in the antileishmanial and antitumor activities of edelfosine, and that both ROS generation and ΔΨ_m_ disruption are involved in edelfosine-induced cell death in *Leishmania* parasites and human cancer cells.

### Effects of respiration inhibitors and uncouplers on edelfosine-induced ROS generation in *Leishmania* and tumor cells

We next examined whether the mitochondrial respiration chain was involved in edelfosine-induced ROS production, by using the following mitochondrial respiration inhibitors: rotenone, complex I inhibitor; malonate, complex II inhibitor; antimycin A, complex III inhibitor; azide, complex IV inhibitor; oligomycin, mitochondrial F_O_F_1_-ATP synthase inhibitor; and carbonyl cyanide *m*-chlorophenyl hydrazone (CCCP), one of the most frequently used uncouplers of oxidative phosphorylation [51]. Incubation of cells with CCCP, which disrupts the proton gradient by carrying protons across the mitochondrial membrane and causes mitochondrial depolarization, prompted the generation of ROS in *L*. *panamensis* and Jurkat cells, and increased edelfosine-induced ROS generation (Fig 5E and 5F). Incubation of *L*. *panamensis* and Jurkat cells for 9 h with rotenone, malonate, antimycin A or azide, affecting the electron transport at specific sites, neither promoted ROS generation nor affected edelfosine-mediated ROS production significantly (Fig 5E and 5F). In contrast, oligomycin, a specific inhibitor of the membranous proton channel (F_O_) of mitochondrial F_O_F_1_-ATP synthase [52], reduced ROS production levels induced by edelfosine in *L*. *panamensis* and Jurkat cells (Fig 5E and 5F). Lower concentrations of oligomycin in *L*. *panamensis* (1 μM) as compared to Jurkat cells (10 μM) were used, because *Leishmania* parasites were very sensitive to higher concentrations of oligomycin, resulting in cytotoxicity. Thus, our data suggest that F_O_F_1_-ATP synthase plays a role in edelfosine-mediated ROS production in both *Leishmania* and tumor cells, a process that eventually leads to cell death.

### Bcl-X_L_ inhibits cell death in *Leishmania* and tumor cells

In order to generalize and further support the role of mitochondria in edelfosine-induced cell death in *Leishmania* parasites and tumor cells, *L*. *infantum* and HeLa cells stably transfected with the expression vectors pX63-Neo (*Leishmania*) and pSFFV-Neo (HeLa), containing the human *bcl-x*_*L*_ open reading frame (pX63-*bcl-x*_*L*_ and pSFFV-*bcl-x*_*L*_, respectively), were used. *L*. *infantum* and HeLa cells transfected with control empty pX63-Neo and pSFFV-Neo plasmids, respectively, were used as controls and behaved similarly to wild-type nontransfected *Leishmania* promastigotes and tumor cells, regarding edelfosine-induced cell death (Table 1). We found that Bcl-X_L_ ectopic expression in *L*. *infantum* promastigotes and HeLa tumor cells inhibited the percentage of hypodiploid cells following edelfosine treatment (Table 1), further supporting the critical role of mitochondria in the induction of apoptosis-like cell death in *Leishmania* and tumor cells treated with edelfosine. The concentration of edelfosine was increased to 40 μM in the case of *L*. *infantum* promastigotes as they were rather resistant to APL treatment [26]. Taken together, these assays support a crucial role of mitochondria in edelfosine-induced cell death in both *Leishmania* spp. parasites and tumor cells. Thus, our results with two different species of *Leishmania*, causing distinct forms of disease, namely *L*. *panamensis* (cutaneous and mucocutaneous leishmaniasis) and *L*. *infantum* (visceral leishmaniasis), converge on the critical role of mitochondria in the killing activity of edelfosine on *Leishmania* parasites.

**Table 1:** Inhibition of apoptosis-like cell death by ectopic expression of Bcl-X_L_ in *L*. *infantum* promastigotes and HeLa tumor cells.

	% Hypodiploid cells	
Cells	Control	Edelfosine
*L*. *infantum* (wild type)	1.9 ± 0.5	25.3 ± 2.1
*L*. *infantum*-pX63	2.1 ± 0.7	27.8 ± 2.5
*L*. *infantum*-Bcl-X_L_	2.0 ± 0.5	11.7 ± 2.0*
HeLa (wild type)	2.8 ± 0.7	35.1 ± 2.7
HeLa-pSFFV	3.3 ± 0.9	38.5 ± 3.2
HeLa-Bcl-X_L_	3.1 ± 0.8	4.1 ± 1.0**

Parasites, wild type and transfected with the empty expression vector pX63 (*L*. *infantum*-pX63) or pX63-*bcl-x*_*L*_ (*L*. *infantum*-Bcl-X_L_), and HeLa cancer cells, wild type and transfected with the empty expression vector pSFFV (HeLa-pSFFV) or pSFFV-*bcl-x*_*L*_ (HeLa-Bcl-X_L_), were incubated with 40 μM (*L*. *infantum*) or 10 μM (HeLa) edelfosine for 24 h, and then the percentage of hypodiploid cells (proportion of cells in the sub-G_1_ cell cycle region) was determined by flow cytometry. Untreated control cells were run in parallel. Data shown are means ± SD of three independent experiments.

Asterisks indicate values that are significantly different from drug-treated cells transfected with the empty vectors pX63 and pSFFV, respectively, at *P*<0.05 (*) and *P*<0.01 (**).

### Lipid raft involvement in the uptake and cytotoxic action of edelfosine in *Leishmania* promastigotes and tumor cells

**Fig 6:**
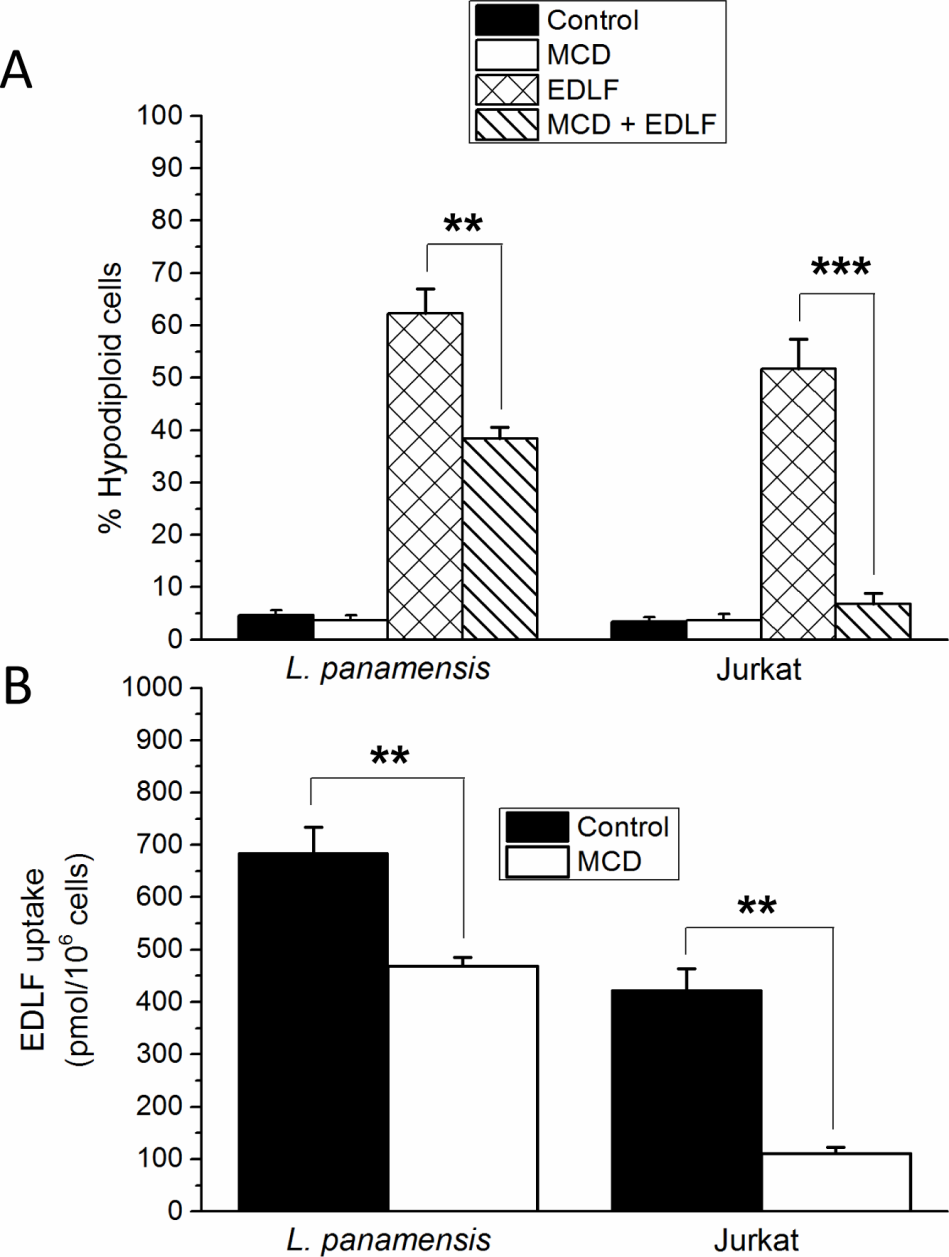
Involvement of lipid rafts in both antileishmanial and anticancer activities and in edelfosine uptake in *Leishmania* promastigotes and cancer cells. (**A**) *L*. *panamensis* promastigotes and T-cell leukemia Jurkat cells were untreated (Control) or pretreated with MCD, and then incubated in the absence or presence of 10 μM edelfosine for 24 h. Percentage of hypodiploid cells were measured by flow cytometry. (**B**) *L*. *panamensis* promastigotes and T-cell leukemia Jurkat cells were untreated (Control) or pretreated with MCD and then incubated with 10 μM [^3^H]edelfosine for 1 h. Drug uptake was determined as shown in the Materials and Methods section. Data shown are means ± SD of three independent experiments performed. Asterisks denote that the differences between the indicated groups are statistically significant. (**) *P*<0.01. (***) *P*<0.001.

Because membrane rafts are a major target in the antitumor action of edelfosine [17–19, 36, 39], we analyzed, in a comparative way, the putative role of lipid rafts in the antileishmanial and anticancer activities of edelfosine. First, we found that raft disruption by preincubation of *L*. *panamensis* promastigotes with the cholesterol-depleting agent MCD [53], led to a partial, but statistically significant, inhibition of both edelfosine-induced cell death (Fig 6A) and edelfosine uptake (Fig 6B), suggesting a role for lipid rafts in *Leishmania* cell death. In addition, cholesterol depletion by MCD strongly inhibited edelfosine-induced cell death and drug uptake in the human T-cell leukemia Jurkat cells (Fig 6A and 6B). It is interesting to note that edelfosine uptake, assessed by the incorporation of [^3^H]edelfosine, was higher in *Leishmania* promastigotes than in tumor cells.

### Mitochondrial F_O_F_1_-ATP synthase is translocated into *L*. *panamensis* and leukemic Jurkat cell lipid rafts and modulates parasite and cancer cell killing upon edelfosine treatment

**Fig 7:**
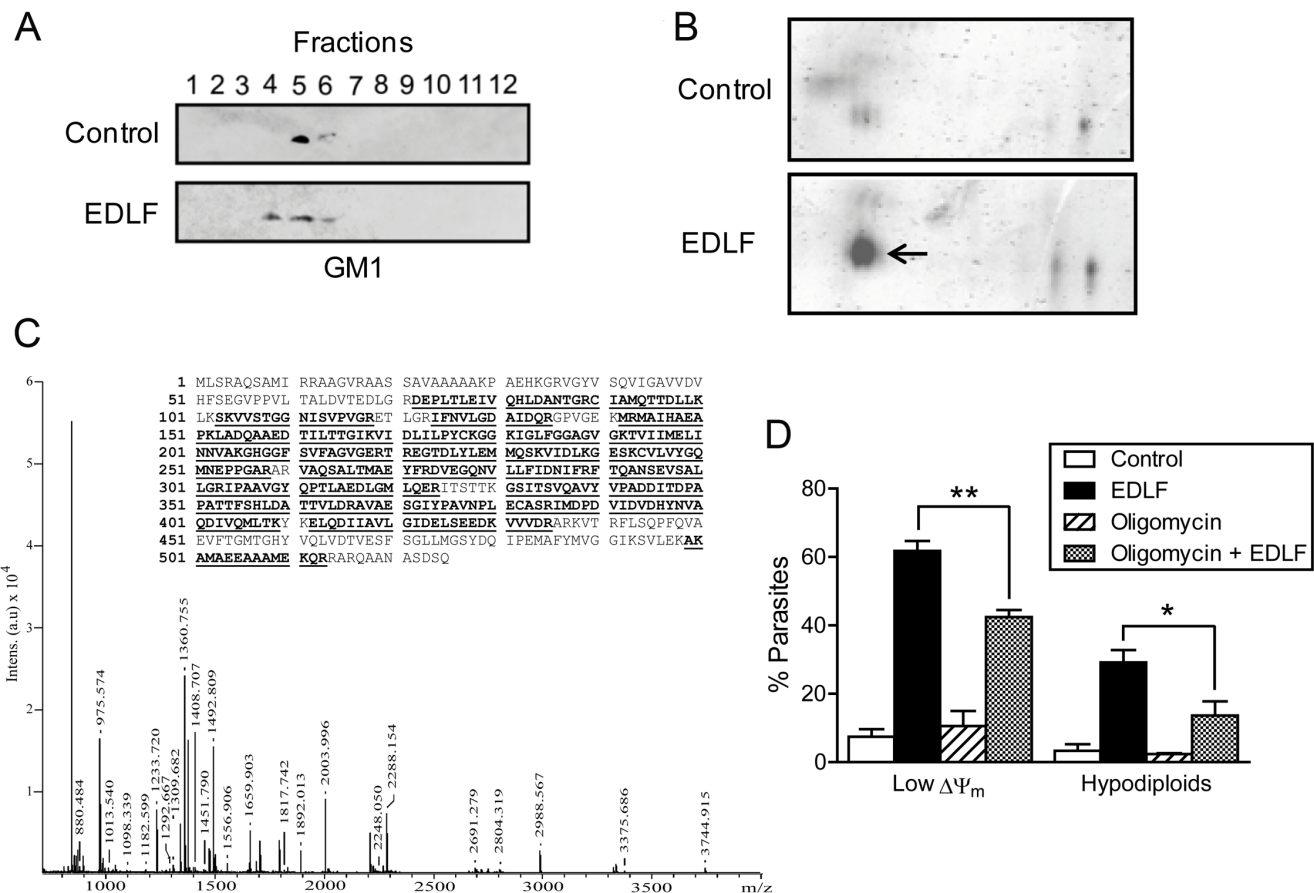
F_O_F_1_-ATPase recruitment into rafts in the antileishmanial activity of edelfosine and oligomycin inhibitory effect on cytotoxicity. (**A**) *L*. *panamensis* promastigotes untreated (Control) and treated with 10 μM edelfosine for 9 h were lysed in 1% Triton X-100 and subjected to discontinuous sucrose density gradient centrifugation. Individual fractions were electrophoresed, and location of GM1 was determined. (**B**) Proteins from lipid rafts of untreated control and edelfosine-treated *L*. *panamensis* promastigotes were subjected to two-dimensional gel electrophoresis followed by MALDI-TOF analysis. Mitochondrial F_O_F_1_-ATP synthase β subunit is indicated by an arrow. (**C**) Mass spectrum of the tryptic peptides of the F_O_F_1_-ATP synthase β subunit spot. Mass values (m/z) and putative amino acid position assignments are indicated above peaks. (*Inset*) Peptide coverage map of *Leishmania* F_O_F_1_-ATP synthase β subunit; the peptides used for identification are highlighted in bold characters and underlined. (**D**) *L*. *panamensis* were untreated (Control) or preincubated with 1 μM oligomycin for 1 h and then incubated in the absence or presence of 10 μM edelfosine for 9 h, and ΔΨ_m_ disruption (Low ΔΨ_m_) and DNA breakdown (hypodiploids) were evaluated. Data shown are means ± SD or representative of three independent experiments. (*) *P*<0.05. (**) *P*<0.01.

The anticancer activity of edelfosine has been shown to depend on the redistribution of lipid raft protein composition [17–19, 36, 38, 39, 54]. Because the above data indicated a remarkable parallelism between the mechanisms of action of edelfosine against *Leishmania* parasites and leukemic cells, we next isolated lipid rafts from untreated and edelfosine-treated *L*. *panamensis* promastigotes by fractionation of low-density detergent-insoluble membranes using discontinuous sucrose density gradient centrifugation. The position of lipid rafts in the sucrose gradients was determined by the presence of ganglioside GM1, detected using the GM1-specific ligand CTx B subunit (Fig 7A), which binds ganglioside GM1 [55], mainly found in rafts [56]. Following a proteomic study of the lipid raft fractions in *L*. *panamensis* promastigotes, we found a dramatic translocation to lipid rafts of mitochondrial F_O_F_1_-ATP synthase β subunit following drug incubation in *L*. *panamensis* promastigotes (Fig 7B and 7C). In addition, we found that oligomycin inhibited edelfosine-induced ΔΨ_m_ disruption and cell death in *Leishmania* (Fig 7D). These data suggest the involvement of F_O_F_1_-ATP synthase and its translocation to lipid rafts in the anti-*Leishmania* activity of edelfosine.

**Fig 8:**
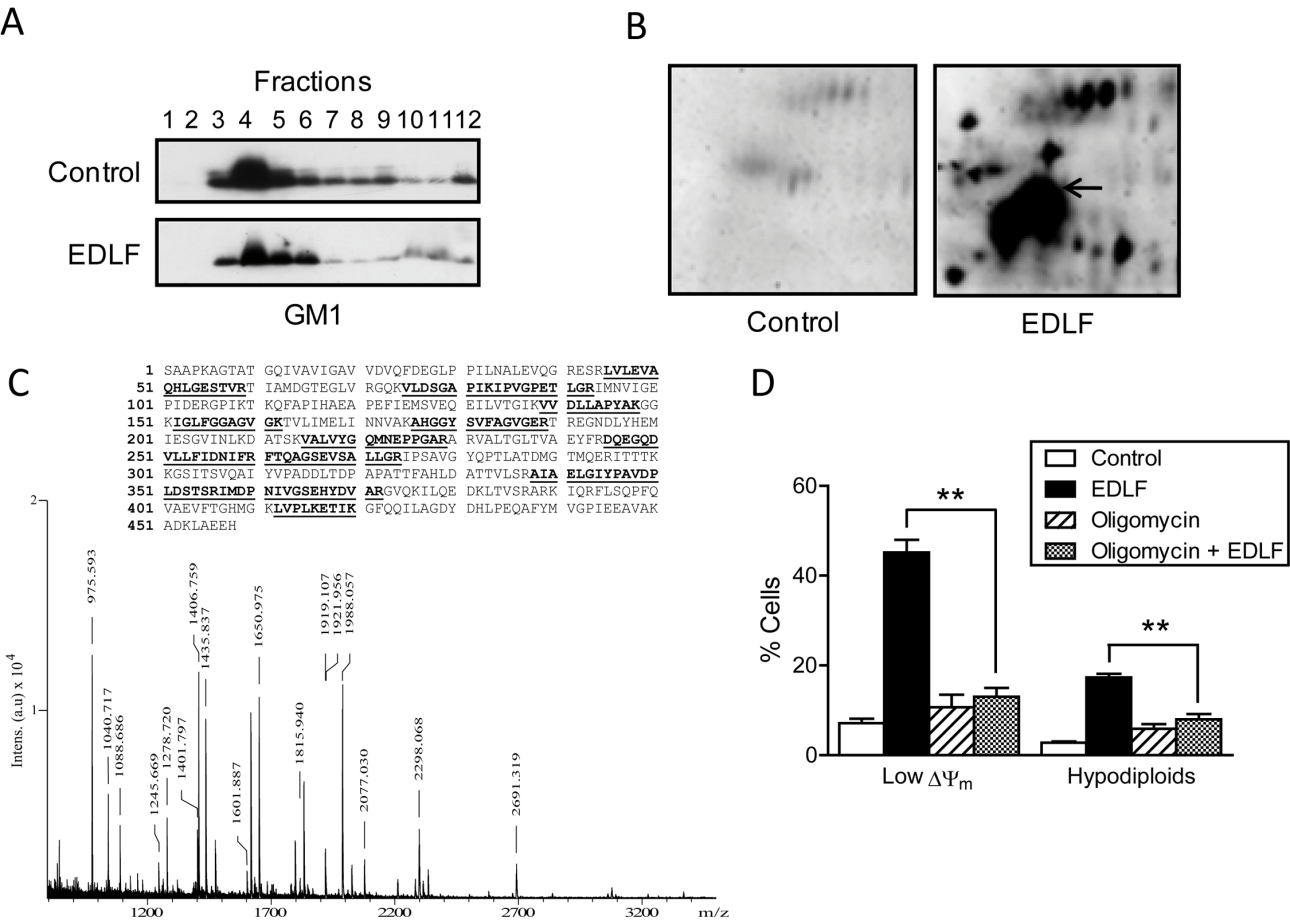
F_O_F_1_-ATPase recruitment in lipid rafts of Jurkat cancer cells after edelfosine incubation and oligomycin inhibitory effect on cytotoxicity. (**A**) Jurkat cells untreated (Control) and treated with 10 μM edelfosine for 9 h were lysed in 1% Triton X-100 and subjected to discontinuous sucrose density gradient centrifugation. Individual fractions were subjected to SDS-PAGE, and location of GM1 was determined using CTx B subunit conjugated with horseradish peroxidase. (**B**) Proteins from lipid rafts of untreated control and edelfosine-treated Jurkat cells were subjected to two-dimensional gel electrophoresis followed by MALDI-TOF analysis. Mitochondrial F_O_F_1_-ATP synthase β subunit is indicated by an arrow. (**C**) Mass spectrum of the tryptic peptides of the F_O_F_1_-ATP synthase β subunit spot. Mass value (m/z) and putative amino acid position assignments are indicated above peaks. (*Inset*) Peptide coverage map of human F_O_F_1_-ATP synthase β subunit; the peptides used for identification are highlighted in bold characters and underlined. (**D**) Jurkat cells were untreated (Control) or preincubated with 10 μM oligomycin for 1 h and then incubated in the absence or presence of 10 μM edelfosine for 9 h, and ΔΨ_m_ disruption (Low ΔΨ_m_) and DNA breakdown (hypodiploids) were evaluated. Data shown are means ± SD or representative of three independent experiments. Asterisks denote that the differences between the indicated groups are statistically significant. (**) *P*<0.01.

We also isolated lipid rafts from untreated and edelfosine-treated human T-cell leukemia Jurkat cells through sucrose gradient centrifugation, and the fractions enriched in lipid rafts were identified using the GM1-specific ligand CTx B subunit (Fig 8A). Similarly to *L*. *panamensis* parasites, a proteomic study of the lipid raft fractions from untreated and drug-treated cancer cells showed a major translocation of mitochondrial F_O_F_1_-ATP synthase β subunit to lipid rafts upon drug incubation in Jurkat cells (Fig 8B and 8C). Furthermore, oligomycin inhibited edelfosine-induced ΔΨ_m_ loss and cell death in Jurkat cells (Fig 8D).

### Genetic abolishment of F_O_F_1_-ATP synthase activity blocks edelfosine cytotoxicity in *Saccharomyces cerevisiae* yeast

Because the above data suggested that F_O_F_1_-ATP synthase could play a major role in the cytotoxic activity of edelfosine against *Leishmania* promastigotes and cancer cells, we next sought to obtain genetic evidence for the role of this enzyme in edelfosine cytotoxicity by using yeast as a eukaryotic model organism. We used *Saccharomyces cerevisiae* yeast *atp7*Δ mutant, with a deletion in the gene encoding for subunit d of the stator stalk of mitochondrial F_O_F_1_-ATP synthase, which is conserved in mammalian cells [57]. Since yeast is a facultative anaerobe and can survive with severely crippled mitochondrial function, we employed *S*. *cerevisiae*, which has been previously shown to be sensitive to edelfosine [58], as a good model for genetic ablation assays. We chose the yeast *atp7*Δ mutant because ATP7 is essential for F_O_F_1_-ATP synthase function, but is not essential for growth in yeast. ATP7 deletion leads to a “petite” phenotype that is slow-growing and unable to survive on nonfermentable carbon sources [57].

**Fig 9:**
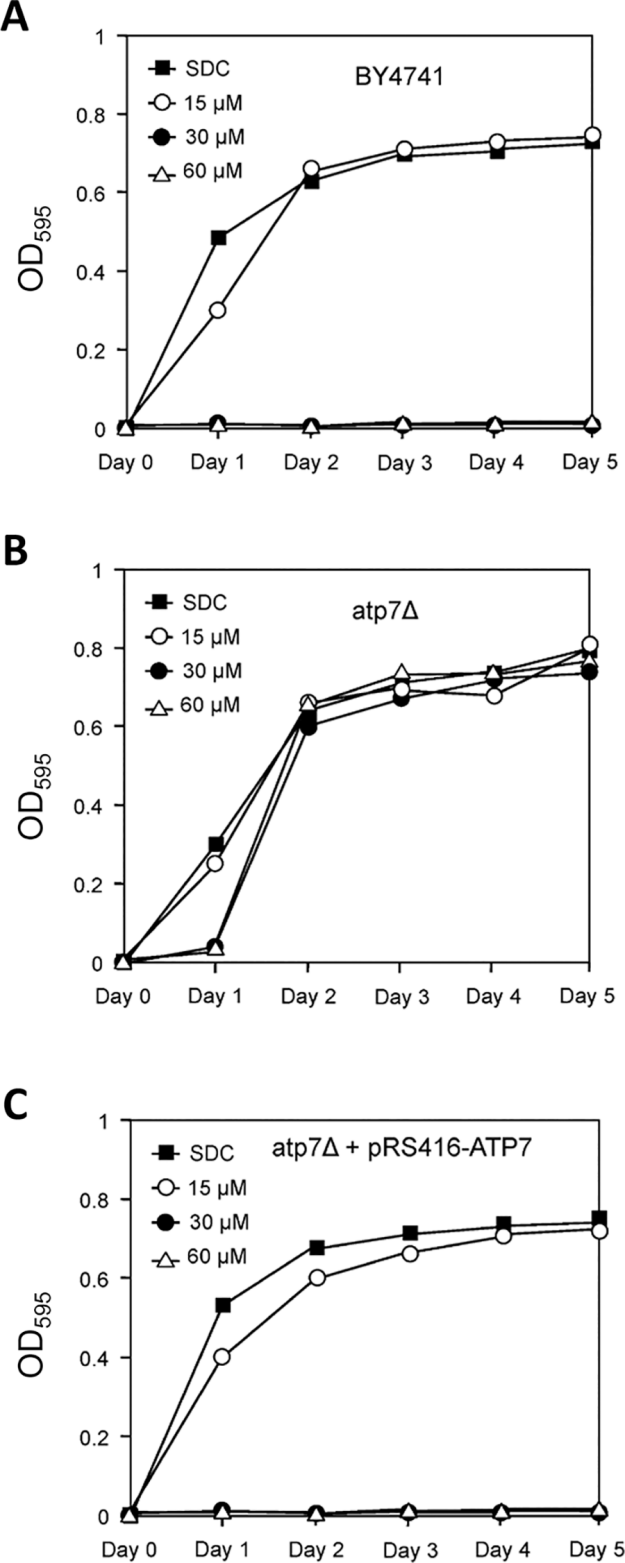
Edelfosine resistance of *atp7*Δ mutant in *Saccharomyces cerevisiae* yeast. Growth curves of wild-type (BY4741) (**A**), *ATP7* knock-out mutant (*atp7*Δ) (**B**) and the mutant strain harboring the corresponding cognate gene (*atp7*Δ+pRS416-*ATP7*) (**C**) in SDC medium containing different concentrations of edelfosine. The cultures were carried out in duplicate and in at least three independent experiments. Data shown are mean values of three independent experiments. SD values were less than 10% of the mean values.

We found that edelfosine inhibited wild-type yeast growth at 30 and 60 μM (Fig 9A), but *atp7*Δ mutant strain was resistant at these drug concentrations (Fig 9B). This edelfosine-resistant phenotype was reverted by transformation of the *atp7*Δ mutant with a centromeric plasmid containing the *atp7* wild-type gene (Fig 9C). Taken together, these results strongly support the involvement of F_O_F_1_-ATPase in the killing activity of edelfosine.

## Discussion

Leishmaniasis therapy is currently far from satisfactory and search for novel druggable targets and new therapeutic approaches is urgently needed. APLs, originally developed as anticancer agents, have proved to show antileishmanial activity, but their mechanisms of action remain to be fully elucidated. Our data reported here indicate that the APL edelfosine is a promising drug against *Leishmania* spp. parasites and tumor cells, and unveil common underlying processes in the killing activity of this APL on both *Leishmania* and cancer cells. Edelfosine killed both *Leishmania* promastigotes and amastigotes by an apoptosis-like process involving DNA breakdown, and edelfosine oral treatment exerted a potent *in vivo* antileishmanial activity. We found here that edelfosine killed intracellular *Leishmania* amastigotes within macrophages, but spared the host cells. Results reported here point out a number of remarkable actions of edelfosine on macrophages, namely: a) normal BMM take up edelfosine, but unlike cancer cells, they are spared; b) edelfosine accumulates in the *Leishmania* amastigotes inside macrophages; c) edelfosine treatment, *in vitro* and *in vivo*, reduces the pro-inflammatory capacity of macrophages. These results suggest that edelfosine kills *Leishmania* parasites by acting directly on the parasite. Edelfosine and other APLs have been shown to act rather selectively on a wide range of malignant cells, mainly due to their predominant uptake by tumor cells [15, 17–19, 43, 59]. The ability of macrophages to take up edelfosine constitutes the first evidence for the incorporation of edelfosine in a normal resting cell type at similar amounts as in cancer cells. This is of major importance in leishmaniasis because macrophages are the main host cells in *Leishmania* infection. We have previously found that the fluorescent edelfosine analog PTE-ET accumulated into naïve macrophages, especially around the nucleus, but once naïve macrophages were infected with *Leishmania* spp., an intense fluorescent signal was detected in the intracellular parasites [26]. These data, together with the findings reported here, indicate that edelfosine is taken up by naïve macrophages in significant amounts, and therefore it might affect some macrophage functions, such as the ones herein described, namely, a decrease in the generation of superoxide anion and nitric oxide, as well as in the production of IL-12+IL-18-induced IFN-γ. Interestingly, when *Leishmania* parasites enter the macrophage, a substantial amount of drug is translocated to the sites where the parasites are located and then the drug is incorporated into the protozoa [26]. Thus, edelfosine could affect both macrophage functions and *Leishmania* survival. The finding that edelfosine diminishes the capacity of macrophages to mount an inflammatory response might be relevant, as severe inflammation at the site of infection leads to tissue destruction in leishmaniasis [60]. In this regard, edelfosine has also been reported to display a potent anti-inflammatory action through L-selectin shedding in human neutrophils, thus preventing neutrophil extravasation [31], and recent *in vivo* evidence further supports the anti-inflammatory and immunomodulatory effect of edelfosine [61–63]. Furthermore, edelfosine promotes apoptosis in mitogen-activated T lymphocytes [64]. On these grounds, edelfosine can affect in different ways the major leukocyte types involved in inflammation, namely neutrophils, macrophages and lymphocytes, thus leading eventually to decreased inflammatory responses.

We have also found here that edelfosine accumulates in mitochondria in both *Leishmania* parasites and tumor cells, leading to ΔΨ_m_ loss and an apoptosis-like cell death. These results agree with recent reports showing a major location of different fluorescent edelfosine analogs in the mitochondria of cancer cells [37, 65]. Our data indicate that edelfosine induces firstly DNA fragmentation in the *Leishmania* kinetoplast-mitochondrion followed by nuclear DNA breakdown, while cell death in *Leishmania* parasites and tumor cells can be inhibited by protecting mitochondria through ectopic Bcl-X_L_ expression. These results indicate a critical role of mitochondria in the edelfosine-induced cell killing mechanism in *Leishmania* parasites and tumor cells. Interestingly, the data reported here suggest that F_O_F_1_-ATP synthase plays a principal role in the edelfosine-induced killing activity in both *Leishmania* parasites and cancer cells. The involvement of the F_O_F_1_-ATP synthase in edelfosine cytotoxicity was further assessed through gene deletion experiments conducted in yeast, by showing that the lack of ATP7, which results in a defective F_O_F_1_-ATP synthase, inhibited edelfosine toxicity. Drug sensitivity was restored when *atp7*Δ mutant yeast were transformed with the cognate wild-type gene. Thus, the results shown here strongly indicate by genetic and biochemical approaches that F_O_F_1_-ATP synthase is involved in the killing activity of edelfosine in both *Leishmania* parasites and human tumor cells.

The major role of mitochondria in edelfosine-induced *Leishmania* killing was further assessed by the generation of ROS in the parasite mitochondrion and the involvement of ROS in edelfosine-induced *Leishmania* promastigote cell death. Interestingly, edelfosine-induced ROS generation in *Leishmania* promastigotes was inhibited by oligomycin, an inhibitor of the F_O_ subunit of the mitochondrial F_O_F_1_-ATP synthase. Taken together, our data suggest a role for mitochondria and ROS generation in the execution of edelfosine-mediated apoptosis, and oligomycin is able to prevent edelfosine-induced ΔΨ_m_ collapse and DNA degradation in both *Leishmania* parasites and cancer cells. These data highlight a major role of the F_O_ component of the F_O_F_1_-ATP synthase in the edelfosine-induced ΔΨ_m_ dissipation, ROS generation and cell death. In this regard, the involvement of F_O_F_1_-ATP synthase in the apoptotic response induced in glioblastoma cells by erucylphosphomocholine (ErPC3, Erufosine), another APL member, has been suggested [66]. Furthermore, oligomycin has also been reported to suppress TNF-induced apoptosis in human epithelioid carcinoma HeLa cells [67].

**Fig 10:**
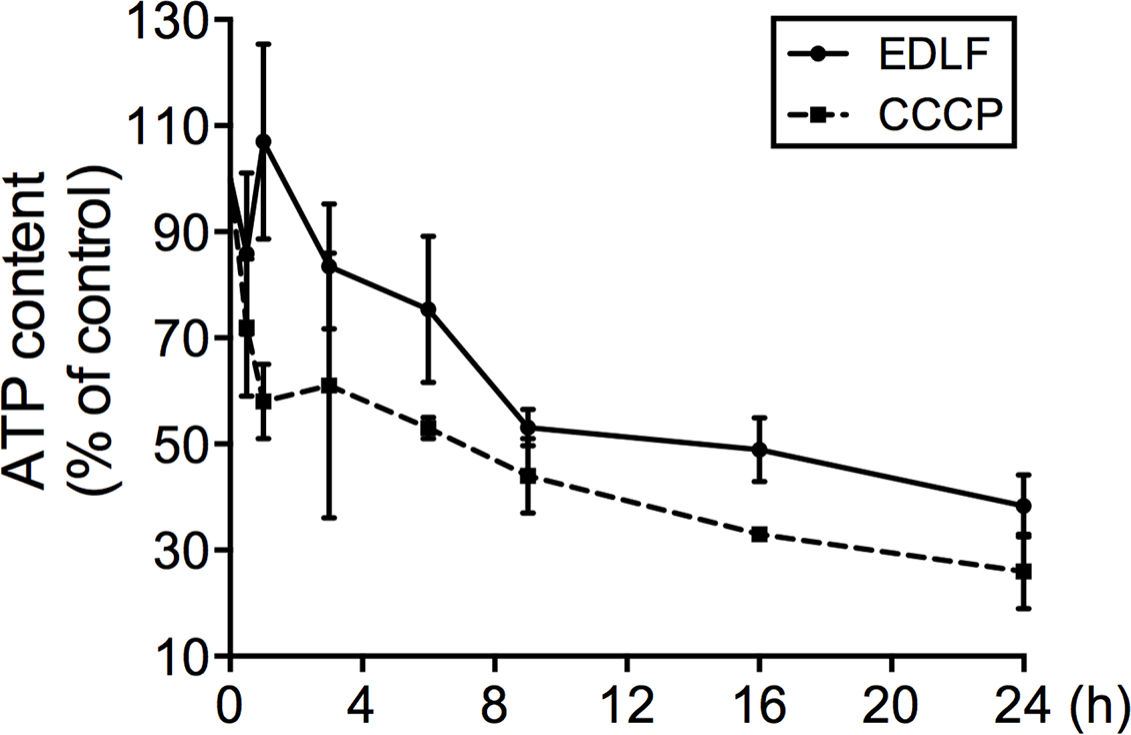
Intracellular ATP measurements. *L*. *panamensis* promastigotes were treated with 10 μM edelfosine or 50 μM CCCP for different incubation times and processed for ATP intracellular content. Results are expressed as percentage of the amount of ATP found in untreated control cells. Data shown are means ± SD of three independent experiments.

The mechanism by which F_O_F_1_-ATPase contributes to edelfosine-induced cell death remains to be established. F_O_F_1_-ATPase resides in the inner membrane of mitochondria and can pump protons in forward and reverse directions, either pumping protons into the mitochondrial matrix, flowing down their concentration gradient and leading to ATP generation, or pumping protons out of the mitochondrial matrix while hydrolyzing ATP. Because edelfosine affects membrane lipid organization, making membranes more fluid [68, 69], it might be suggested that edelfosine makes the outer membrane more porous, thus favoring the leakage of H^+^ ions from the outer-inner intermembrane space into the cytosol, which leads to the dissipation of the proton gradient. As a consequence, the F_O_F_1_-ATP synthase could run in reverse, that is, hydrolyzing ATP and alkalinizing the matrix by proton extrusion. Because matrix alkalinization has been shown to cause opening of the mitochondria permeability transition pore [70], the F_O_F_1_-ATP synthase could facilitate cell death by this mechanism. This explanation has been previously proposed for the effect of oligomycin in inhibiting Bax-induced apoptosis in yeast and mammalian cells [71]. In this regard, we have found that edelfosine treatment led to a reduction in the ATP content of *L*. *panamensis* promastigotes (Fig 10).

Furthermore, edelfosine has been reported to act through lipid rafts in human leukemic cancer cells [17–19, 22, 23, 36, 39], and recent evidence suggests a raft-mediated connection between the cell membrane and mitochondria in the action of edelfosine [20, 37, 38]. Here, we have found the involvement of lipid rafts in the antileishmanial activity of edelfosine, and edelfosine treatment led to a dramatic recruitment of mitochondrial F_O_F_1_-ATP synthase into rafts in both *Leishmania* promastigotes and cancer cells. These findings are in line with the identification of lipid rafts in *Leishmania* parasites [24], and with the impairment of miltefosine action against *L*. *donovani* by membrane sterol depletion [72]. The results reported here suggest a redistribution of the F_O_F_1_-ATP synthase within the mitochondria or to additional raft domains in other cellular membranes following edelfosine treatment, thus altering the normal function of the enzyme and affecting cell viability. It could also be envisaged that the action of edelfosine on lipid rafts and mitochondria might underlie the inhibition of superoxide anion production in edelfosine-treated macrophages, generated by the NADPH-oxidase located at the macrophage cell membrane [73], and the enhancement of mitochondria-dependent ROS generation in drug-sensitive cells.

**Fig 11:**
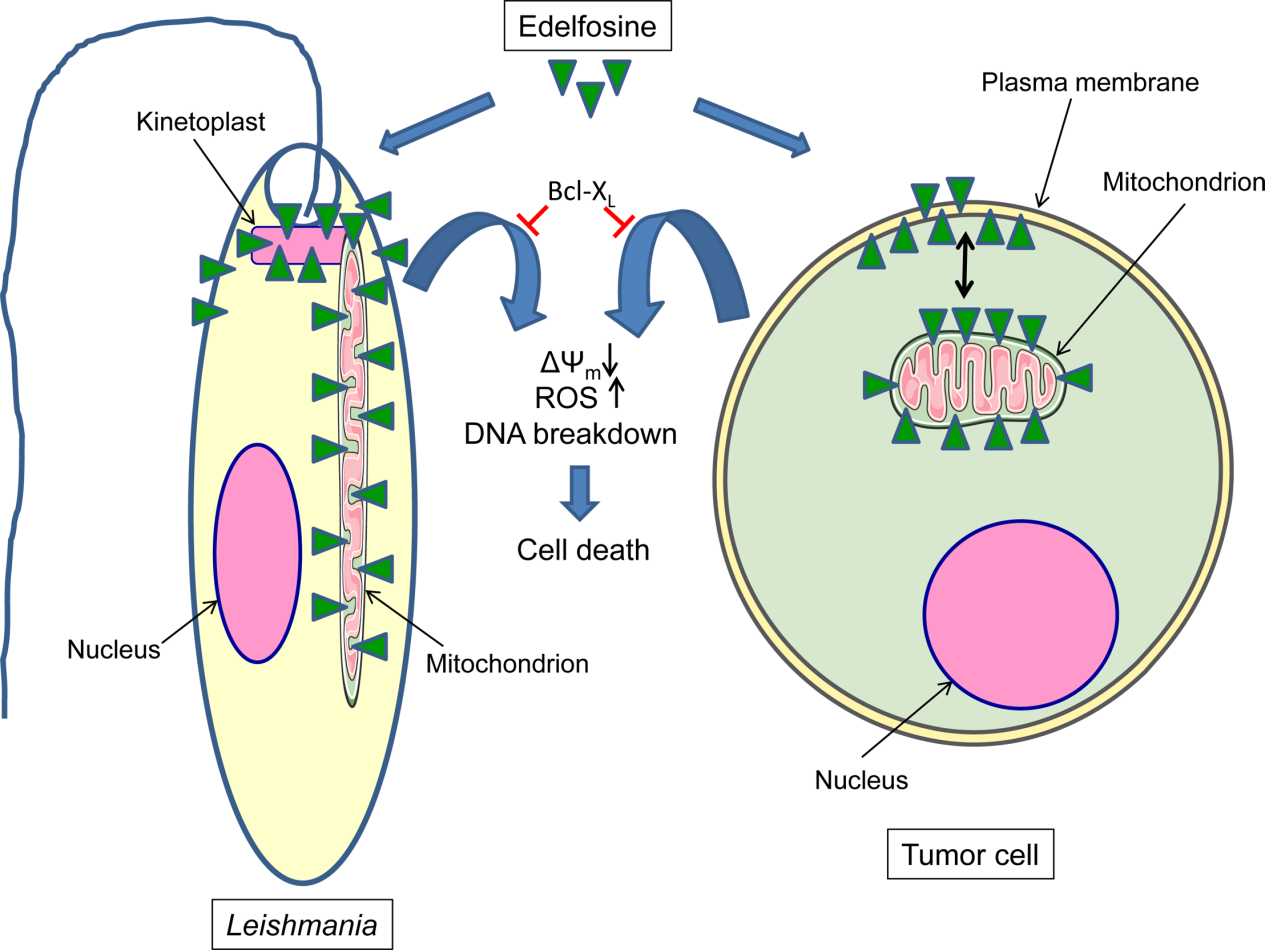
Schematic model of mitochondria involvement in the killing activity of edelfosine against *Leishmania* parasites and tumor cells. This is a schematic diagram to portray one currently plausible mechanism of how edelfosine induces cell death in *Leishmania* parasites and tumor cells through its main mitochondrial localization in both biological systems. Protection of mitochondria by Bcl-X_L_ ectopic expression restrains cell death. See text for details.

The results reported here highlight a major role for mitochondria and lipid rafts in the mechanism of action of edelfosine as both antileishmanial and anticancer drug. Nevertheless, cancer cells seem to be more dependable on lipid rafts than parasites, as shown by the relatively higher inhibition observed in drug uptake and drug-induced cell death when rafts were disrupted by sterol depletion (Fig 6). This putative mechanism of action involving mitochondria, and briefly depicted in Fig 11, seems to be common to both *Leishmania* parasites and tumor cells. The fact that protecting mitochondria by Bcl-X_L_ ectopic expression leads to an inhibition in drug-induced cell death, further supports the major role of mitochondria and mitochondrial-mediated pathways in the killing activity of edelfosine in both *Leishmania* parasites and human cancer cells. Previous data on human cancer cells have demonstrated the involvement of mitochondria in the pro-apoptotic activity of edelfosine as an antitumor drug [16, 18, 20, 37, 38, 46, 74], and the results reported here extrapolate this notion to its leishmanicidal activity. In addition, the present results pinpoint the major role of F_O_F_1_-ATPase in the killing activity of edelfosine against *Leishmania* parasites and tumor cells. We have previously found a link between lipid rafts and mitochondria in the mechanism of action of edelfosine [37, 38], suggesting an edelfosine-mediated redistribution of lipid rafts from the plasma membrane to mitochondria [37, 38]. The results reported here indicate that F_O_F_1_-ATPase is either translocated to cell surface lipid rafts or to raft domains present in mitochondria. A number of reports have shown the presence of raft-localized F_O_F_1_-ATP synthase at the cell surface of several cell types, having been proposed to act as a receptor for different ligands, a proton channel, or a modulator of extracellular ATP levels, involved in numerous biological processes through still unclear mechanisms [75–81]. Our results cannot discern between a cell surface and a mitochondrial localization for the raft-associated F_O_F_1_-ATP synthase following edelfosine treatment reported here. Thus, a putative translocation of the mitochondrial F_O_F_1_-ATP synthase to the cell surface cannot be ruled out at the moment, and additional experimental approaches should be applied to elucidate the prevailing localization of raft-located F_O_F_1_-ATP synthase. However, our present data indicating an accumulation of the ether lipid in the mitochondria of both *Leishmania* parasites and cancer cells, lead us to suggest that a plausible mechanism could involve the translocation of edelfosine from the plasma membrane to the mitochondria where it would ultimately exert its cytotoxic activity promoting the accumulation of F_O_F_1_-ATP synthase into mitochondrial rafts, and leading to the dissipation of the mitochondrial membrane potential, ROS generation, and eventually cell demise (Fig 11). The fact that kinetoplast-mitochondrion was the first organelle where ROS metabolites were generated and DNA was broken down, preceding nuclear DNA fragmentation, points out the critical role of mitochondria as a major target in the search for effective drugs to treat leishmaniasis. It is tempting to suggest that a link between lipid rafts and mitochondria could lead to interesting hints to unveil a novel framework in both *Leishmania* and cancer therapy. The present data also indicate that our insight on how edelfosine works as an antitumor drug can be of great aid to and give valuable information to uncover the mechanism of action of its leishmanicidal activity, which could be hypothetically extrapolated to other antileishmanial drugs, and might be of inspiration to further identify potential common therapeutic targets in cancer and leishmaniasis.

Taken together, our data indicate that the edelfosine antileishmanial and antitumor mechanisms of action share similar molecular processes, involving mitochondria, lipid rafts and F_O_F_1_-ATPase. This study provides a molecular explanation on how the antitumor drug edelfosine acts as an antileishmanial agent, and highlights that mitochondria, lipid rafts and F_O_F_1_-ATPase act as major players in cell death modulation, opening new avenues for therapeutic intervention in leishmaniasis and cancer. Our results show that the ether phospholipid edelfosine can be a promising orally administered therapeutic agent and a lead compound in the search for novel and much-needed antileishmanial agents, and identify lipid rafts, mitochondria and F_O_F_1_-ATPase as appealing new antileishmanial targets. Furthermore, the results shown here indicate that edelfosine is very effective in killing different species of *Leishmania* parasites, as well as in distinct developmental stages, such as promastigotes and amastigotes. Interestingly, recent data have shown an increasing rate of relapse against miltefosine and a decline in its efficacy [82–85], which could correspond to the readiness in acquiring experimental drug resistance to miltefosine *in vitro* [86–88]. We have previously shown that edelfosine is less prone to lead to drug resistance development than miltefosine, and displays a higher antileishmanial activity than miltefosine against a wide variety of *Leishmania* spp. [26]. Thus the potent leishmanicidal activity of edelfosine, together with its low toxicity profile [31], warrants further clinical evaluation for this ether lipid as a possible therapeutic agent against different forms of leishmaniasis.
